# Effects of camelina oil supplementation on lipid profile and glycemic control: a systematic review and dose‒response meta-analysis of randomized clinical trials

**DOI:** 10.1186/s12944-022-01745-4

**Published:** 2022-12-07

**Authors:** Cyrus Jalili, Sepide Talebi, Sanaz Mehrabani, Reza Bagheri, Alexei Wong, Parsa Amirian, Mahsa Zarpoosh, Seyed Mojtaba Ghoreishy, Mohammad Ali Hojjati Kermani, Sajjad Moradi

**Affiliations:** 1 Medical Biology Research Center, Health Technology Institute, Kermanshah University of Medical Sciences, Kermanshah, Iran; 2 Department of Clinical Nutrition, School of Nutritional Science, Tehran University of Medical Science, Tehran, Iran; 3 Department of Clinical Nutrition, School of Nutrition and Food Science, Isfahan University of Medical Sciences, Isfahan, Iran; 4 Department of Exercise Physiology, University of Isfahan, Isfahan, Iran; 5 Department of Health and Human Performance, Marymount University, Arlington, VA USA; 6 General Practitioner, Kermanshah University of Medical Sciences (KUMS), Kermanshah, Iran; 7 Clinical Tuberculosis and Epidemiology Research Center, National Research Institute of Tuberculosis and Lung Diseases (NRITLD), Masih Daneshvari Hospital, Shahid Beheshti University of Medical Sciences, Tehran, Iran; 8 Nutritional Sciences Department, School of Nutritional Sciences and Food Technology, Kermanshah University of Medical Sciences, Kermanshah, Iran

**Keywords:** Camelina oil, cardiovascular, lipid profile, meta-analysis

## Abstract

**Background:**

This systematic review and dose–response meta-analysis of published randomized controlled trials (RCTs) was conducted to determine the effectiveness of camelina oil supplementation (COS) on lipid profiles and glycemic indices.

**Methods:**

Relevant RCTs were selected by searching the ISI Web of Science, PubMed, and Scopus databases up to July 1, 2022. RTCs with an intervention duration of less than 2 weeks, without a placebo group, and those that used COS in combination with another supplement were excluded. Weighted mean differences and 95% confidence intervals were pooled by applying a random-effects model, while validated methods examined sensitivity analyses, heterogeneity, and publication bias.

**Results:**

Seven eligible RCTs, including 428 individuals, were selected. The pooled analysis revealed that COS significantly improved total cholesterol in studies lasting more than 8 weeks and utilizing dosages lower than 30 g/d compared to the placebo group. The results of fractional polynomial modeling indicated that there were nonlinear dose–response relations between the dose of COS and absolute mean differences in low-density cholesterol, high-density cholesterol, and total cholesterol, but not triglycerides. It appears that the greatest effect of COS oil occurs at the dosage of 20 g/day.

**Conclusion:**

The present meta-analysis indicates that COS may reduce cardiovascular disease risk by improving lipid profile markers. Based on the results of this study, COS at dosages lower than 30 g/d may be a beneficial nonpharmacological strategy for lipid control. Further RCTs with longer COS durations are warranted to expand on these results.

**Supplementary Information:**

The online version contains supplementary material available at 10.1186/s12944-022-01745-4.

## Introduction

Prior research indicates that alpha-linolenic acid (18:3, n-3; ALA) can reduce the risk of cardiovascular disease (CVD) by improving blood lipids, blood pressure, and hemostatic factors, among others [1–3]. According to a meta-analysis published in 2020, an increase in the intake of ALA is associated with a decrease in triglycerides (TGs), total cholesterol (TC), low-density cholesterol (LDL), and very low-density lipoprotein cholesterol (VLDL) levels [1]. Camelina oil (derived from *Camelina sativa*), a lesser-known oil, is considered a good source of ALA compared to other edible oils; 36 to 40% of its fatty acid content is ALA, an n-3 fatty acid derived from plants [4, 5]. Moreover, it is one of the richest dietary sources of omega-3 fatty acids, with a polyunsaturated fatty acid (PUFA) content over 50%, as well as high contents of antioxidants, namely, tocopherols (55.8–76.1 mg/100 g), carotenoids (103–198 mg of carotene/kg), and phytosterols (331–442 mg/100 g) [6, 7].

Several randomized controlled trials (RCTs) were conducted to evaluate the efficacy of camelina oil on CVD-related markers, including lipid profile and glycemic parameters [6–12]. These investigations have yielded contradictory results. For instance, Musazadeh 2021 et al. [7] and Bellien et al. [8] revealed that COS might attenuate glycemic parameters in nonalcoholic fatty liver disease (NAFLD) and hypertensive patients, respectively. However, Schwab et al. [12] showed that COS did not affect glycemic parameters among participants with impaired fasting glucose. Moreover, Musazadeh et al. [6] showed that COS improved the lipid profile in NAFLD patients. Camelina oil has been suggested to modulate fatty acid synthesis and oxidation through the upregulation of β-oxidation gene expression, such as peroxisome proliferator-activated receptor α (PPARα) and carnitine palmitoyltransferase-1 (CPT-1). Furthermore, it has also been proposed to inhibit lipogenic gene expression, such as sterol regulatory element binding proteins (SREBPs), carbohydrate-responsive element-binding protein and PPARγ [6]. However, some studies did not show any significant effects of COS on the lipid profile as a CVD-related marker [8, 12]. Thus, research on this topic has shown mixed findings, leading to a lack of consensus on the impact of COS on lipid profiles and glycemic control. There are currently no investigations to systematically assess and summarize findings on this topic, representing a knowledge gap. Therefore, a systematic review and meta-analysis of published RCTs was conducted to determine the effectiveness of COS on lipid profiles and glycemic control in human studies.

### Experimental methods

#### Systematic search and study selection

The study’s protocol was registered in the International Prospective Register of Systematic Reviews Database (CRD42021275655) and conducted according to the 2020 PRISMA guidelines [13]. An explanation of the population, intervention, comparator, and outcome (PICO) framework is reported in Supplementary Table 1. A systematic literature search was performed in ISI Web of Science, Scopus, the Cochrane Library databases, and the PubMed search engine up to November 26, 2022, with no date or language limitations. The search strategy and the key terms are illustrated in Supplementary Table 2. A supplementary literature search was extended to Google Scholar with the screening of camelina oil-related terms up to November 26, 2022. The first ten pages of all search records were scanned. Database searches were completed in conjunction with the bibliographical examination of all relevant papers. Two authors (separately) performed the systematic screening. Any disagreements were resolved by discussion with another researcher.

#### Eligibility Criteria

Two researchers screened the titles, abstracts, and full texts of relevant studies. All RCTs in humans (either parallel or crossover designs) that evaluated the effect of COS on the lipid profile (LDL, HDL, TG, and TC) and glycemic indices (FBS and FI) were selected. The exclusion criteria were as follows: (1) clinical trials with an intervention duration of less than 2 weeks, (2) clinical trials without a placebo group and those that were not randomized, (3) use of COS in combination with another supplement, (4) observational or animal studies, book section, editorial, conference paper, letter, short survey, notes and (4) those with insufficient data for the outcomes of interest.

#### Data extraction

The main features of the included studies are reported in Table 1. If there were no available relevant data, corresponding authors were contacted to obtain any missing data. The data extraction procedure was conducted separately by two researchers to ensure reliability. Any disagreements were resolved by consensus and discussion.

**Table 1:** Main characteristics of included studies

First author (publication year)	Country	Sample size (Intervention/Control) Sex	Target Population	Mean Age (Intervention/Control)	Mean BMI (Intervention/Control)	RCT design (Blinding)	Duration (weeks)	Form and dose of intervention	Comparison	Results
Karvonen et al. 2002 [10]	Finland	68 (23/45) Male and female	Hypercholesterolemic subjects	NR (28 to 65 years)	NR	Parallel (Double)	6 Weeks	Camelina oil 30 g/d	Canola oil 30 g/d or olive oil	Camelina oil consumption did not significantly change in lipid profile comparison with placebo use.
Schwab et al. 2018 [12]	Finland	43 (23/21) Male and female	Volunteers with impaired fasting glucose	58.9 ± 6.5	29.2 ± 2.4	Parallel (Double)	12 Weeks	Camelina oil 10 g/d	Limited intakes of fish and source of alpha-linolenic acid	Camelina oil consumption did not significantly change in glucose metabolism parameters comparison with placebo use.
Musazadeh et al. 2021 [7]	Iran	130 (63/67) Male and female	NAFLD patients	44.30 ± 4.38/ 43.86 ± 6.07	34.19 ± 3.55/ 33.90 ± 2.65	Parallel (Triple)	12 Weeks	Camelina oil 20 g/d	Sunflower oil 20 g/d	Camelina oil consumption causes significant changes in lipid profile (except HDL-c) among NAFLD patients
Dobrzyńska et al. 2021 [9]	Poland	60 (30/30) Female	Postmenopausal women with dyslipidaemia	55 ± 5/ 57 ± 4	26.7 ± 5.3/ 26.9 ± 6.4	Parallel (Double)	6 Weeks	Camelina oil 30 g/d	Canola oil 30 g/d	Camelina oil consumption did not change in lipid profile, anthropometric parameters and blood pressure comparison with placebo use.
*Lankinen* et al. *2021* [11]	Finland	46 (23/23) Male	Healthy men	66.6 ± 5.6/ 64.8 ± 5.6	24.6 ± 2.6/ 24.8 ± 2.6	Parallel (None)	8 Weeks	Camelina oil 50 ml/d	Sunflower oil 50 ml/d	Camelina oil consumption did not significantly change in FBS comparison with placebo use.
Musazadeh et al. 2022 [6]	Iran	130 (63/67) Male and female	NAFLD patients	44.30 ± 4.38/ 43.86 ± 6.07	34.19 ± 3.55/ 33.90 ± 2.65	Parallel (Triple)	12 Weeks	Camelina oil 20 g/d	Sunflower oil 20 g/d	Camelina oil intake led to a significant decrease in FI, and HOMA-IR in compared with the placebo group.
Bellien et al. 2022 [8]	France	81 (40/41) Male and female	Hypertensive patients with metabolic syndrome	57.7 ± 8.4/ 55.0 ± 7.5	30.0 ± 4.0/ 30.3 ± 4.2	Parallel (Double)	24 Weeks	Camelina oil 5.2 g/d	A mixture of cyclodextrin	Compared with placebo, camelina oil increased fasting glycemia and HOMA-IR index, without affecting plasma lipids

*NR* Not reported, *HDL* High-density lipoprotein, *FBS* Fasting blood sugar, *NAFLD* Nonalcoholic fatty liver disease, *FI* Fating insulin, *HOMA-IR* Homeostasis model assessment of insulin resistance

#### Quality assessment of studies

The Cochrane Collaboration tool [14] was applied to assess the quality of articles according to the following criteria: (1) random sequence generation (selection bias), (2) allocation concealment (selection bias), (3) blinding (performance bias and detection bias), (4) separation for blinding of participants and personnel, as well as blinding of outcome assessment, (5) incomplete outcome data (attrition bias), (6) selective reporting (reporting bias), and (7) other biases (any important concerns about bias not covered in the other domains of the tool). Each area was categorized into three levels: low risk of bias (bias, if present, is unlikely to alter the results seriously), high risk of bias (bias may alter the results seriously), and unclear risk of bias (a risk of bias that raises some doubt about the results). Based on these areas, the overall quality of each study was weighed as good (low risk for more than two items), fair (low risk for two items), and weak (low risk for less than two items) [15].

#### Meta-analysis of data

To assess the effect size of the lipid and glycemic markers, the mean and standard deviation (SD) changes were extracted from the COS and placebo groups. Subgroup analyses relating to the study duration (≤ 8 weeks and > 8 weeks), sex (female or both), body mass (normal, overweight, or obese), dosage (< 30 g/day and ≥ 30 g/day), participants’ baseline body mass index (≤ 25 and > 25) and mean age (≤ 55 and > 55 years) were carried out to identify potential sources of heterogeneity. For the random-effects model, the DerSimonian and Laird method was applied [16]. Within-group changes were calculated by subtracting the baseline mean from the final mean value in each group. The SD of the mean difference was calculated using the following formula:

$\textrm{SD}\ \textrm{change}=\sqrt{\left[{\left(\textrm{SD}\ \textrm{baseline}\right)}^2+{\left(\textrm{SD}\ \textrm{final}\right)}^2-\left(2\times 0.8\times \textrm{SD}\ \textrm{baseline}\times \textrm{SD}\ \textrm{final}\right)\right]}$ [17]. For trials that reported only the standard error of the mean (SEM), SD was calculated by applying the following formula: SD = $SEM\ x\ \sqrt{n}$, where “n” represents the number of participants in each group. Heterogeneity between studies was evaluated by Cochrane’s Q test (significance at *P* < 0.100) and the I^2^ index. The potential nonlinear effects of COS dose (g/d) and study duration (week) were evaluated by applying fractional polynomial modeling [18]. Sensitivity analysis was performed by removing each study one by one and recalculating the pooled assessments. Publication bias was evaluated by Egger’s regression asymmetry [19]. All statistical analyses were performed utilizing STATA software, version 16 (Stata Corp LP, College Station, TX). The results were considered significant at *P* < 0.05.

#### Certainty assessment

The general certainty of evidence in randomized clinical trials was ranked utilizing the Grading of Recommendations Assessment, Development, and Evaluation (GRADE) working group guidelines. According to the relevant evaluation criteria, the quality of evidence was ranked into four classes: high, moderate, low, and very low [20].

## Results

### Selection and identification of studies

**Fig. 1:**
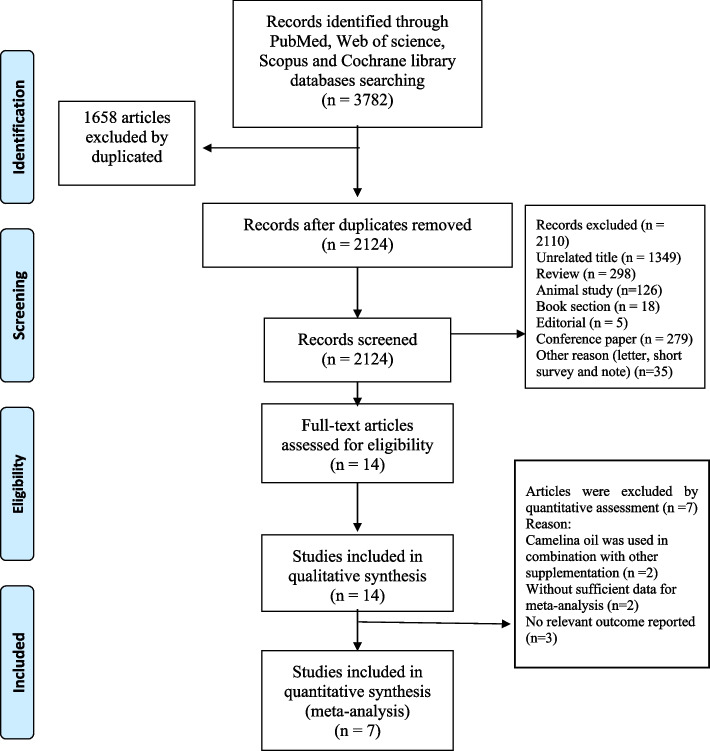
PRISMA flowchart describing the study’s systematic literature search and study selection

The study’s systematic literature search and study selection flow are reported in Fig. 1. The systematic literature search found a total of 3782 studies, of which 2124 were evaluated (1658 articles excluded by duplication). Two thousand one hundred ten records did not meet the inclusion criteria and were excluded from qualitative and quantitative analyses. In contrast, seven studies were excluded from the quantitative evaluation for reporting irrelevant outcomes (*n* = 3), not presenting sufficient data (*n* = 2), and using Camelina oil in combination with other supplementations (*n* = 2) (Supplementary Table 3). Finally, seven RCTs, collectively comprising six markers, were identified for the quantitative analysis [6–12].

### Characteristics of studies

The seven eligible RCTs included 428 individuals (202 participants in the COS group and 226 in the control group) **(**Table 1**)**. The mean age of the participants ranged from 44.30 ± 4.38 to 66.6 ± 5.6 years. Trials were conducted in Finland [10–12], Iran [6, 7], Poland [11] and France [8]. The included clinical trials were conducted in healthy men [11] and hypercholesterolemic participants [10], as well as in participants with impaired fasting glucose [12], nonalcoholic fatty liver disease participants [6, 7], postmenopausal women with dyslipidemia [9], and hypertensive patients with metabolic syndrome [8]. All of the studies applied a parallel arm setting. These articles were published between 2002 and 2022. The dose of COS used ranged from 50 ml/d to 30 g/d, while the length of the interventions ranged from 6 to 24 weeks. The types of interventions used for the control groups included canola oil [9, 10], sunflower oil [6, 7, 11], a diet with limited intake of fish and sources of ALA [12], and a mixture of cyclodextrin [8].

### Quality assessment of studies

Based on the results of the Cochrane risk of bias tools, all clinical trials were categorized as good quality (demonstrating a low risk of bias on ≥3 domains) [6–12] **(**Table 2**)**. As illustrated in Table 2**,** all studies were ranked low risk for random sequence generation and allocation concealment domains [6–12]. Lankinen et al. [11] was ranked at high risk for blinding participants and personnel and blinding of the outcome assessors’ domains. However, these bias domains were low risk for other studies [6–10, 12]. The incomplete outcome data domain of bias was low risk for six studies [6–9, 11, 12], and the Karvonen et al. study [10] was ranked as having an unclear risk of bias. Four studies were ranked low risk [6, 8, 11, 12], and three were categorized as having an unclear risk for the selective reporting bias domain [7, 9, 10]. All studies were ranked as low risk for other bias domains [6–12].

**Table 2:** Quality assessment by the Cochrane Collaboration’s tool

Study	Random Sequence Generation	Allocation concealment	Blinding of participants personnel	Blinding of outcome assessors	Incomplete outcome data	Selective outcome reporting	Other sources of bias
Karvonen et al. 2002 [10]	+	+	+	+	?	?	+
Schwab et al. 2018 [12]	+	+	+	+	+	+	+
Musazadeh et al. 2021 [7]	+	+	+	+	+	?	+
Dobrzyńska et al. 2021 [9]	+	+	+	+	+	?	+
*Lankinen* et al. *2021* [11]	+	+	–	–	+	+	+
Musazadeh et al. 2022 [6]	+	+	+	+	+	+	+
Bellien et al. 2022 [8]	+	+	+	+	+	+	+

Legend: + = Low risk of bias, − = High risk of bias,? = Unclear risk of bias

### Meta-analysis of data

#### Effects of COS on glycemic indices

As shown in Table 3, pooled data from four clinical trials demonstrated that the COS did not change FBG (− 1.86 mg/dl; 95% CI: − 6.77, 3.06; I^2^ = 89.0%; *P* = 0.459) or FI (− 0.10 pmol/L; 95% CI: − 0.72, 0.52; I^2^ = 81.1%; *P* = 0.752) compared to the placebo group (Supplemental Figs. 1 and 2, Part A).

**Table 3:** Summery effects of Camelina oil on cardiovascular risk factors

Outcomes (unit)	Effect sizes (N)	Participants (n)		WMD (95% CI)^a^	***P***-***value***	I^**2** b^ (%)	P^c^_**heterogeneity**_	Egger
		Camelina oil	Placebo					
**Glycemic indices**								
FBG (mg/dl)	(4)	118	113	−1.86 (− 6.77, 3.06)	0.459	89.0	< 0.001	0.970
FI (pmol/L)	(4]	118	113	−0.10 (−0.72, 0.52)	0.752	81.1	0.001	0.275
**Blood lipids**								
LDL-C (mg/dl)	(6)	155	156	−3.16 (−7.40, 1.09)	0.145	0.0	0.583	0.128
HDL-C (mg/dl)	(4)	109	111	0.41 (−2.27, 3.12)	0.763	0.0	0.775	0.200
TC (mg/dl)	(6)	155	156	−4.06 (−9.46, 1.34)	0.141	3.1	0.397	0.117
TG (mg/dl)	(4)	109	111	−4.92 (−19.59, 9.76)	0.512	31.5	0.223	0.610

*FBG* Fasting blood glucose, *FI* Fasting insulin, *HDL-C* High density cholesterol, *HOMA-IR* Homeostasis model assessment-estimated insulin resistance, *LDL-C* Low density cholesterol, *TC* Total cholesterol, *TG* Triglycerides

^a^Obtained from random effects model

^b^Inconsistency—percentage of variation across studies due to heterogeneity

^c^Obtained from fixed effects model

#### Effects of COS on lipid profile

**Fig. 2:**
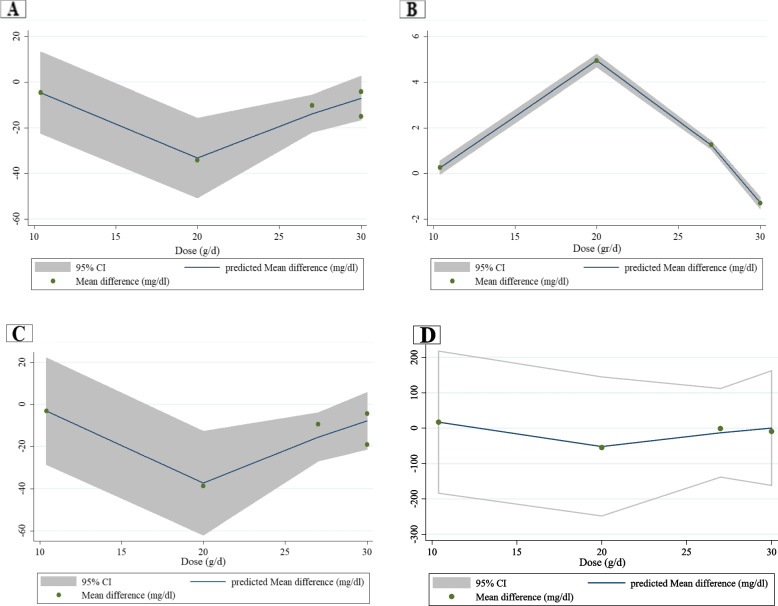
Non-linear dose-response relations between dose of Camelina oil intervention and absolute mean differences in lipid profile, A: LDL, B: HDL, C: Total cholesterol, and D: Triglyceride

**Fig. 3:**
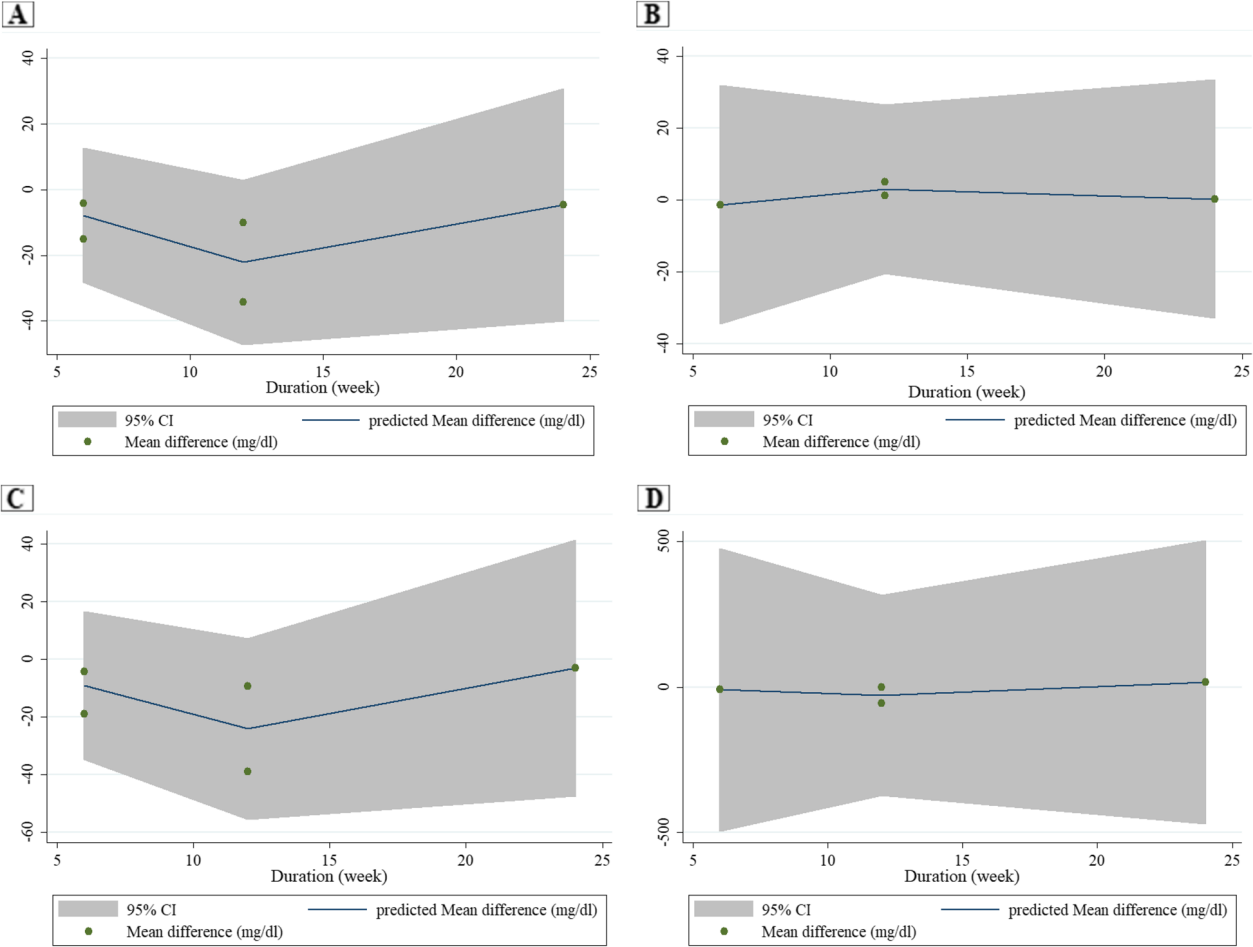
Non-linear dose-response relations between duration of Camelina oil intervention and absolute mean differences in lipid profile, A: LDL, B: HDL, C: Total cholesterol, and D: Triglyceride

Data analysis from six trials that evaluated the lipid profile showed that COS did not significantly change LDL (− 3.16 mg/dl; 95% CI: − 7.40, 1.09; I^2^ = 0.0%; *P* = 0.145; *n* = 6), HDL (0.41 mg/dl; 95% CI: − 2.27, 3.12; I^2^ = 0.0%; *P* = 0.763), TC (− 4.06 mg/dl; 95% CI: − 9.46, 1.34; I^2^ = 3.1%; *P* = 0.141) or TG (− 4.92 mg/dl; 95% CI: − 19.59, 9.76; I^2^ = 31.5%; *P* = 0.512) compared to the control group (Table 3 and Supplemental Figs. 3 to 6, Part A). However, subgroup analysis according to duration and the dose of the intervention showed that COS significantly decreased TC (− 11.64 mg/dl; 95% CI: − 25.49, − 2.21; I^2^ = 35.8%; *P* = 0.009) **(**Table 4**)** in trials with more than 8 weeks in length and dosages of less than 30 g/d. The results of fractional polynomial modeling indicated that there were nonlinear dose–response relations between the dose of COS and absolute mean differences in LDL (*P* = 0.024), HDL (*P* = 0.003), and TC (*P* = 0.042) but not TG (*P* = 0.515) **(**Fig. 2**)**. According to Fig. 2, the greatest COS effect occurs at a dosage of 20 g/day. However, there were no relationships between the duration of COS intervention and absolute mean differences in LDL (*P* = 0.250), HDL (*P* = 0.532), TC (*P* = 0.276), and TG (*P* = 0.515) **(**Fig. 3**)**.

**Table 4:** Result of subgroup analysis of included studies in meta-analysis

Sub-grouped by	No. of trials	Effect size^a^	95% Confidence interval, ***P*** value	I^**2**^ (%)	P for heterogeneity	P for between subgroup heterogeneity
**Low density lipoprotein cholesterol**						
Duration						0.237
≤ 8 weeks	3	−1.79	(−6.60, 3.03), 0.467	0.0	0.974	
> 8 weeks	3	−8.43	(−18.77, 1.92), 0.110	13.9	0.313	
Dose						0.237
< 30 g/day	3	−1.79	(−6.60, 3.03), 0.467	0.0	0.974	
≥ 30 g/day	3	−8.43	(−18.77, 1.92), 0.110	13.9	0.313	
Sex						0.937
Female	1	−4.00	(−25.40, 17.40), 0.714	–	–	
Both	5	−3.12	(−7.46, 1.21), 0.439	0.0	0.437	
Baseline BMI						0.362
≤ 25	2	−1.67	(−6.61, 3.27),	0.0	0.923	
> 25	2	−5.22	(−14.89, 4.45),	0.0	0.833	
Age						0.638
≤ 55 y	3	−3.65	(−11.05, 3.76), 0.335	42.8	0.174	
> 55 y	3	−5.01	(−13.83, 3.80), 0.265	0.0	0.973	
**Total cholesterol**						
Duration						0.163
≤ 8 weeks	3	−1.57	(−7.82, 4.68), 0.622	0.0	0.949	
> 8 weeks	3	−11.64	(−25.49, −2.21), 0.009	35.8	0.211	
Dose						0.163
< 30 g/day	3	−11.64	(− 25.49, − 2.21), 0.009	35.8	0.211	
≥ 30 g/day	3	−1.57	(−7.82, 4.68), 0.622	0.0	0.949	
Sex						0.925
Female	1	−5.00	(−27.04, 17.04), 0.657	–	–	
Both	5	−4.54	(−11.06, 1.97), 0.172	22.3	0.272	
Baseline BMI						0.230
≤ 25	2	−1.27	(−7.79, 5.25), 0.702	0.0	0.958	
> 25	2	−6.00	(−16.61, 4.62), 0.268	0.0	0.986	
Age						0.652
≤ 55 y	3	−5.69	(−16.85, 5.46), 0.317	59.6	0.084	
> 55 y	3	−5.81	(−15.37, 3.76), 0.234	0.0	0.997	

^a^Calculated by Random-effects model

#### Sensitivity Analysis

Sensitivity analysis was conducted by removing each of the selected trials. The outcomes revealed that the weighted mean difference (WMD) was not altered remarkably by removing each of the trials. This showed that the meta-analysis outcomes were stable and not sensitive to any of the seven trials.

### Publication Bias

Furthermore, no evidence of publication bias was observed for the effect of COS on FBS (*P* = 0.970, Egger’s test), FI (*P* = 0.275, Egger’s test), LDL (*P* = 0.128, Egger’s test), HDL (*P* = 0.128, Egger’s test), TG (*P* = 0.200, Egger’s test), or TC (*P* = 0.117, Egger’s test) **(**Table 1**).** In addition, the funnel plots were symmetrical, which showed no clear publication bias among the included studies (Supplemental Figs. 1 to 6, Part B).

### Quality of evidence

The GRADE guidelines were utilized to assess the quality of evidence for the outcomes. The effects of LDL, HDL, TG, and TC were downgraded to a moderate level. Moreover, FBS and FI were categorized as very low quality **(**Table 5**)**.

**Table 5:** GRADE profile of collagen peptide supplementation for cardiovasscular risk factors scores in adults

Quality assessment						Summary of findings				Quality of evidence
Outcomes	Risk of bias	Inconsistency	Indirectness	Imprecision	Publication Bias	Participants (n)		WMD (95%CI)	Heterogeneity (I^2^)	
						Camelina	Placebo			
FBG	No serious limitations	Very serious Limitations	Serious Limitations	Serious Limitations	No serious limitations	118	113	−1.86 (−6.77, 3.06)	89.0%	⊕◯◯◯ Very low
FI	No serious limitations	Very serious Limitations	Serious Limitations	Serious Limitations	No serious limitations	118	113	−0.10 (−0.72, 0.52)	81.1%	⊕◯◯◯ Very low
LDL-C	No serious limitations	No serious limitations	No serious limitations	Serious Limitations	No serious limitations	155	156	−4.06 (−9.46, 1.34)	3.1%	⊕ ⊕ ⊕◯ Moderate
HDL-C	No serious limitations	No serious Limitations	No serious limitations	Serious limitations	No serious limitations	109	111	−4.92 (−19.59, 9.76)	31.5%	⊕ ⊕ ⊕◯ Moderate
TC	No serious limitations	No serious Limitations	No serious limitations	Serious Limitations	No serious limitations	155	156	−3.16 (−7.40, 1.09)	0.0%	⊕ ⊕ ⊕◯ Moderate
TG	No serious limitations	No serious Limitations	No serious limitations	Serious Limitations	No serious limitations	109	111	0.41 (−2.27, 3.12)	0.0%	⊕ ⊕ ⊕◯ Moderate

*FBG* Fasting blood glucose, *FI* Fasting insulin, *HDL-C* High density cholesterol, *HOMA-IR* Homeostasis model assessment-estimated insulin resistance, *LDL-C* Low density cholesterol, *TC* Total cholesterol, *TG* Triglycerides

## Discussion

The current meta-analytic investigation assessed the effects of COS on lipid and glycemic profiles. Pooled data analysis did not show any effects of COS on lipid profile and glycemic indices compared with placebo intake. However, subgroup analysis showed that COS for more than 8 weeks and at a dose lower than 30 g/d could decrease TC. Furthermore, the results indicated that there were nonlinear dose–response relations between the dose of COS and absolute mean differences in LDL, HDL, and TC, but not TG. The greatest COS effect occurs at a dosage of 20 g/day.

Recently, nutraceutical products have gained attention for reducing the risk of CVD. This is important, as data showed 18.6 million deaths due to CVD in 2019 alone [21]. *Camelina sativa* L., also known as false flax, may have a beneficial effect on reducing CVD risk due to its high content of polyunsaturated fatty acids. Because omega-3 fatty acids are abundant in Camelina oil and contain 40–45% ALA, 15% linoleic acid (LA), and a low amount of saturated fatty acids (SFAs) (approximately 6%) [22], it is theorized that COS could improve CVD risk. However, no study has summarized previous findings on this topic. The current investigation revealed a cardioprotective impact of COS through a systematic review and meta-analysis for the first time.

The analysis revealed no effect of COS on FBG and FI. In line with these results, a study performed on participants with impaired glucose metabolism found that 12 weeks of COS intervention did not affect glycemic control [12]. Additionally, another study showed that an 8-week COS intervention did not change fasting glucose and FI levels compared with sunflower oil consumption [11]. Another study in NAFLD participants demonstrated that COS intake for 12 weeks did not alter FBG, but the intervention improved fasting insulin concentration in comparison with 20 g/d sunflower oil intake [7]. In contrast with the results of this study, a recent clinical trial conducted by Bellien et al. among hypertensive patients with metabolic syndrome compared the effects of cyclodextrin-complexed camelina oil with a placebo containing cyclodextrins and wheat starch for 6 months and demonstrated that COS intake enhanced fasting glycemia [8]. Since the Bellien study was longer than prior investigations, it seems that long-term COS intake can alter glucose metabolism, and these inconsistent results in the available literature may be due to the different durations of COS interventions. Genetic factors can also affect fatty acid composition. For instance, delta-5-desaturase and delta-6 desaturase are limiting enzymes in the endogenous pathway of omega-3, and omega-3 biosynthesis is encoded by fatty acid desaturase-1 and fatty acid desaturase-2 genes. Therefore, variation and single nucleotide polymorphisms of fatty acid desaturase-1 and fatty acid desaturase-2 genes can affect the biosynthesis of PUFAs [23]. Moreover, a study by Lankinen et al. in participants with different FADS1 rs174550 genotypes (TT or CC) revealed that COS intake for 8 weeks increased fasting glucose levels in men with the carrier TT genotype for *FADS1* rs174550 compared to baseline values [11]. Additionally, a recent meta-analysis revealed that omega-3 intake increases the gluconeogenesis of glycerol [24]. Consequently, chronic high-dose omega-3 treatment could negatively affect glycemic control among diabetes mellitus patients [24]. Another meta-analysis of 8 clinical trials in type 2 diabetes found that ALA-enriched diets with a median of 4.4 g/d ALA did not change FBG or FI [25]. There is evidence showing that the conversion rate of ALA to eicosapentaenoic acid (EPA) and docosahexaenoic acid (DHA) is low, so the beneficial effect of EPA/DHA derived from ALA intake on the glycemic profile is doubtful [26, 27]. On the other hand, in some included studies, sunflower oil was considered a placebo for COS intake. Sunflower oil encompasses nearly 85% unsaturated fatty acids (14–43% oleic and 44–75% linoleic acids) [28]. The beneficial effect of oleic acid intake on the glycemic profile was observed previously. A prior investigation showed that oleic acid simplifies the uptake of glucose in adipocyte tissue by enhancing the signaling of insulin receptors [29]. Furthermore, meta-analytic work by Wu et al. [30] demonstrated that a higher ratio of linoleic acid biomarkers was related to a reduced risk of type 2 diabetes, which might be related to increased insulin sensitivity [31]. Thus, it seems that if studies considered another component as a placebo, the favorable effects of COS could be better manifested. Moreover, the lack of significant changes in markers may be related to the small number of studies. Hence, more RCTs are warranted to further assess glycemic indices following COS, particularly using placebo interventions that have no favorable effect on glycemic control as well as including participants with insulin resistance.

Dyslipidemia is another risk factor for CVD that plays an important role in the initiation and progression of the disease. The pooled data analysis did not show a significant change in the lipid profile after COS compared to placebo. Karvonen et al. [10] conducted a clinical trial comparing the cholesterol-lowering effects of Camelina, rapeseed, and olive oil (30 g/d for each) in hypercholesterolemic participants. Their findings revealed that cholesterol levels decreased in all three groups compared with baseline values [10]. However, they did not find a significant difference between the three groups. This study used COS at a dose of 30 g/d for a short-term duration. It seems that a longer duration of dietary intervention may be needed to exert more noticeable cholesterol-lowering effects. The findings of this investigation also showed that ALA (C18:3, n-3) increased significantly in the camelina oil compared to the other groups. It seems that using other oils as a placebo could not be effective in finding between-group differences because rapeseed oil and olive oil have a cholesterol-lowering effect [32–34]. Rapeseed oil contains polyphenols and high amounts of unsaturated fatty acids, mainly monounsaturated fatty acids, that can effectively reduce cholesterol levels by enhancing the excretion of bile acid and reducing cholesterol absorption [32]. Moreover, olive oil contains approximately 55–83% oleic acid, 4–20% PUFA and other components, such as phenolic compounds [35].

A recent meta-analysis revealed that olive oil intake led to a decrease in TG, TC and LDL-C, but its effects were lower than those of other vegetable oils, including omega-3-rich vegetable oils. However, prior research indicates that refined olive oil could not exert a beneficial effect on the lipid profile [36]. This may be due to the higher level of antioxidants and the existence of phytochemical composites in virgin olive oil compared with refined olive oil [35]. The beneficial effect of olive oil on TG levels may also be due to its high amount of MUFAs. It has been shown that MUFAs reduce TG by affecting the enzymes that are involved in the metabolism of VLDL-C [37]. Furthermore, a recent study conducted in postmenopausal women with dyslipidemia revealed that there were no differences between the COS and canola oil groups in terms of lipid profile. However, TC, LDL-C, TG and non-HDL-C decreased in both groups after the six-week intervention compared with their baseline values [9]. This study also used canola oil for comparison, which itself has beneficial effects on the lipid profile. A recent meta-analysis found that canola oil intake led to decreased LDL-C, TC and LDL-C/HDL-C ratios compared with olive oil intake [38]. Both of these oils contain high amounts of MUFAs, but canola oil contains more PUFAs, particularly ALA [39–41]. The exact mechanisms for the lipid-lowering effects of canola oil have not yet been determined, but they may be due to its fatty acid components. ALA can reduce the activity of the limiting enzyme in cholesterol synthesis, β-hydroxy β-methylglutaryl-CoA (HMG-CoA) [42, 43]. Additionally, it can play a role in increasing the beta oxidation of fatty acids in the mitochondria, which can lead to decreases in both TG synthesis and the activity of enzymes involved in fatty acid synthesis [44–46]. Another recent study in hypertensive patients with metabolic syndrome did not demonstrate any beneficial effects on the lipid profile after 6 months of COS intake (10.4 g/d) when compared to a placebo intervention. However, 50% of participants in that study consumed lipid-lowering agents, which may have affected their results [8]. In contrast, Scwab et al. showed that 30 ml COS for 12 weeks improved LDL and TC compared with groups that consumed fatty fish and lean fish but not in comparison with groups that were instructed to limit intake of fish and ALA sources [12]. Additionally, Musazadeh et al. revealed that COS decreased TC, LDL, and TG in nonalcoholic fatty liver disease (NAFLD) patients after a 12-week intervention compared with a placebo that contained sunflower oil, although HDL did not change [6]. According to the subgroup results, TC decreased in doses of less than 30 g/d and intervention durations of more than 8 weeks. It is plausible that COS higher than 30 g/d can lead to an increase in the percentage of energy intake, as a previous study demonstrated that ALA intake higher than eight g/d increased energy intake and consequently increased the risk of metabolic syndrome [47, 48].

### Study strengths and limitations

The main strength of the current study is that it is the first meta-analysis study that assessed the effects of COS as a nutraceutical component on indicators of CVD risk. A limitation of the present research is the small number of studies included in the analysis. This may have played a role in the lack of significant changes in some of the assessed parameters. It is essential that more studies be conducted in this field. Additionally, studies were performed in Iran and European countries. Further studies in other areas are needed to determine whether outcomes apply to other ethnic cohorts.

## Conclusion

The present meta-analysis showed that COS improved TC in studies lasting more than 8 weeks and dosages lower than 30 g/d. Decreases of 39 mg/dL in TC values can diminish all-cause and coronary heart disease-related mortalities by 24 and 25%, respectively [49]. Thus, the declines in TC (− 11.64 mg/dl) concentrations revealed by our analysis support the clinical significance of COS as a nonpharmacological strategy for the improvement of this lipid marker. In addition, the results of fractional polynomial modeling indicated that there were nonlinear dose–response relations between the dose of COS and absolute mean differences in LDL, HDL, and TC but not TG. The greatest COS effect occurs at a dosage of 20 g/day. Based on the results of this study, hypercholestrolemic participants may benefit from long-term consumption of this oil at a dosage of less than 30 g/d, and it may be considered adjuvant therapy for them; however, more studies are needed to confirm this finding. According to the data pooled in this study, some investigations used different types of oils as a placebo. These oils can have beneficial effects on lipid profiles and glycemic control, which may have affected the results of those investigations. Consequently, it is recommended to design further studies with a suitable placebo. Additional studies utilizing different dosages and populations are recommended to expand the current findings.

## Supplementary Information

**Additional file 1: Supplemental Table 1.** Description of population, intervention, comparator and outcome (PICO).

**Additional file 2: Supplementary Table 2.** Search strategies including the key terms and the queries for each database.

**Additional file 3: Supplemental Table 3.** Reason for exclusion of retrieved articles.

**Additional file 4: Supplemental Fig. 1.** Forest plot (A) and funnel plot (B) of effect of camelina oil supplementation on fasting blood glucose. **Supplemental Fig. 2.** Forest plot (A) and funnel plot (B) of effect of camelina oil supplementation on fasting insulin. **Supplemental Fig. 3.** Forest plot (A) and funnel plot (B) of effect of camelina oil supplementation on low-density cholesterol. **Supplemental Fig. 4.** Forest plot (A) and funnel plot (B) of effect of camelina oil supplementation on high-density cholesterol. **Supplemental Fig. 5.** Forest plot (A) and funnel plot (B) of effect of camelina oil supplementation on triglycerides. **Supplemental Fig. 6.** Forest plot (A) and funnel plot (B) of effect of camelina oil supplementation on total cholesterol.

**Additional file 5.**

## Data Availability

The datasets used and/or analyzed during the current study are available as supplementary SPSS.sav format files.

## Abbreviations

COS: Camelina oil supplementation
LDL: Low-density cholesterol
HDL: High-density lipoprotein
TC: Total cholesterol
TG: Triglycerides
FBG: Fasting blood glucose
FI: Fasting insulin
RCTs: Relevant randomized, placebo-controlled trials
WMD: Weighted mean differences
