# Supplementary material for: Effects of camelina oil supplementation on lipid profile and glycemic control: a systematic review and dose‒response meta-analysis of randomized clinical trials
Source: Lipids Health Dis. 2022 Dec 7;21:132. doi: 10.1186/s12944-022-01745-4 (PMC9727906; doi:10.1186/s12944-022-01745-4)
Supplement: Supplementary file 1 — Additional file 1: Supplemental Table 1. Description of population, intervention, comparator and outcome (PICO). [file 12944_2022_1745_MOESM1_ESM.doc]

**Supplemental Table 1** Description of population, intervention, comparator and outcome (PICO)

| Population | Healthy and patients subjects |
| --- | --- |
| Intervention | Camelina oil supplementation |
| Comparison | Placebo or different oils |
| Outcome | Lipid profile (LDL, HDL, triglyceride, and total cholesterol), and glycemic indices (fasting blood sugar, fasting insulin, and insulin resistance) |
