# Supplementary material for: Effects of camelina oil supplementation on lipid profile and glycemic control: a systematic review and dose‒response meta-analysis of randomized clinical trials
Source: Lipids Health Dis. 2022 Dec 7;21:132. doi: 10.1186/s12944-022-01745-4 (PMC9727906; doi:10.1186/s12944-022-01745-4)
Supplement: Supplementary file 2 — Additional file 2: Supplementary Table 2. Search strategies including the key terms and the queries for each database. [file 12944_2022_1745_MOESM2_ESM.docx]

| **Supplementary Table 2.** Search strategies including the key terms and the queries for each database | |
| --- | --- |
| **Database** ( up to  July 1, 2022) | **Key terms and the queries** |
| PubMed  (n=490) | "Camelina sativa" [tiab] OR "Camelina" [tiab] OR "Gold-of-pleasure" [tiab] OR "False flax" [tiab] OR "Wild flax" [tiab] OR "German sesame" [tiab] OR "Siberian oilseed" [tiab] |
| ISI Web of Science  (n=1613) | "Camelina sativa” (All Fields) or "Camelina” (All Fields) or "Gold-of-pleasure” (All Fields) or "False flax” (All Fields) or "Wild flax” (All Fields) or "German sesame" (All Fields) or "Siberian oilseed" (All Fields) |
| Scopus  (n=1644) | ( TITLE-ABS-KEY ( "Camelina sativa" ) OR TITLE-ABS-KEY ( "Camelina" ) OR TITLE-ABS-KEY ( "Gold-of-pleasure" ) OR TITLE-ABS-KEY ( "False flax" ) OR TITLE-ABS-KEY ( "Wild flax" ) OR TITLE-ABS-KEY ( "German sesame" ) OR TITLE-ABS-KEY ( "Siberian oilseed" ) ) |
| Cochrane library(n=35) | "Camelina sativa” OR "Camelina” OR "Gold-of-pleasure” OR "False flax” OR "Wild flax” OR "German sesame" OR "Siberian oilseed" |
