# Supplementary material for: Effects of camelina oil supplementation on lipid profile and glycemic control: a systematic review and dose‒response meta-analysis of randomized clinical trials
Source: Lipids Health Dis. 2022 Dec 7;21:132. doi: 10.1186/s12944-022-01745-4 (PMC9727906; doi:10.1186/s12944-022-01745-4)
Supplement: Supplementary file 3 — Additional file 3: Supplemental Table 3. Reason for exclusion of retrieved articles. [file 12944_2022_1745_MOESM3_ESM.docx]

| **Supplemental Table 2**: Reason for exclusion of retrieved articles | |
| --- | --- |
| References | Reason for exclusion |
| 1. Kavyani M, Saleh-Ghadimi S, Dehghan P, Khoshbaten M. Co-supplementation of camelina oil and a prebiotic is more effective for in improving cardiometabolic risk factors and mental health in patients with NAFLD: a randomized clinical trial. Food & Function. 2021 Aug 2. 2. Farhangi MA, Dehghan P, Musazadeh V, Kavyani M, Maleki P. Effectiveness of omega-3 and prebiotics on adiponectin, leptin, liver enzymes lipid profile and anthropometric indices in patients with non-alcoholic fatty liver disease: A randomized controlled trial. Journal of Functional Foods. 2022 May 1;92:105074. | Camelina oil was used in combination with other supplementation |
| 1. Meuronen T, Lankinen MA, Fauland A, Shimizu BI, de Mello VD, Laaksonen DE, Wheelock CE, Erkkilä AT, Schwab US. Intake of camelina sativa oil and fatty fish alter the plasma lipid mediator profile in subjects with impaired glucose metabolism–A randomized controlled trial. Prostaglandins, Leukotrienes and Essential Fatty Acids. 2020 Aug 1;159:102143. 2. Erkkilä AT, Manninen S, Fredrikson L, Bhalke M, Holopainen M, Ruuth M, Lankinen M, Käkelä R, Öörni K, Schwab US. Lipidomic changes of LDL after consumption of Camelina sativa oil, fatty fish and lean fish in subjects with impaired glucose metabolism—A randomized controlled trial. Journal of Clinical Lipidology. 2021 Sep 1;15(5):743-51. | Without sufficient data |
| 1. Manninen S, Lankinen M, Erkkilä A, Nguyen SD, Ruuth M, de Mello V, Öörni K, Schwab U. The effect of intakes of fish and Camelina sativa oil on atherogenic and anti-atherogenic functions of LDL and HDL particles: A randomized controlled trial. Atherosclerosis. 2019 Feb 1;281:56-61. 2. Rahman MJ, Ambigaipalan P, Shahidi F. Biological activities of camelina and sophia seeds phenolics: inhibition of LDL oxidation, DNA damage, and pancreatic lipase and α‐glucosidase activities. Journal of food science. 2018 Jan;83(1):237-45 3. West AL, Miles EA, Han L, Lillycrop KA, Napier JA, Calder PC, Burdge GC. Dietary Supplementation with Transgenic Camelina sativa Oil Containing 20: 5n-3 and 22: 6n-3 or Fish Oil Induces Differential Changes in the Transcriptome of CD3+ T Lymphocytes. Nutrients. 2021 Sep 5;13(9):3116. | No relevant outcome reported |
