# Supplementary material for: Effects of camelina oil supplementation on lipid profile and glycemic control: a systematic review and dose‒response meta-analysis of randomized clinical trials
Source: Lipids Health Dis. 2022 Dec 7;21:132. doi: 10.1186/s12944-022-01745-4 (PMC9727906; doi:10.1186/s12944-022-01745-4)
Supplement: Supplementary file 4 — Additional file 4: Supplemental Fig. 1. Forest plot (A) and funnel plot (B) of effect of camelina oil supplementation on fasting blood glucose. Supplemental Fig. 2. Forest plot (A) and funnel plot (B) of effect of camelina oil supplementation on fasting insulin. Supplemental Fig. 3. Forest plot (A) and funnel plot (B) of effect of camelina oil supplementation on low-density cholesterol. Supplemental Fig. 4. Forest plot (A) and funnel plot (B) of effect of camelina oil supplementation on high-density cholesterol. Supplemental Fig. 5. Forest plot (A) and funnel plot (B) of effect of camelina oil supplementation on triglycerides. Supplemental Fig. 6. Forest plot (A) and funnel plot (B) of effect of camelina oil supplementation on total cholesterol. [file 12944_2022_1745_MOESM4_ESM.docx]

**Supplemental Figure 1:** Forest plot (A) and funnel plot (B) of effect of camelina oil supplementation on fasting blood glucose

| **Supplemental Figure 1: A** | **Supplemental Figure 1: B** |
| --- | --- |
| 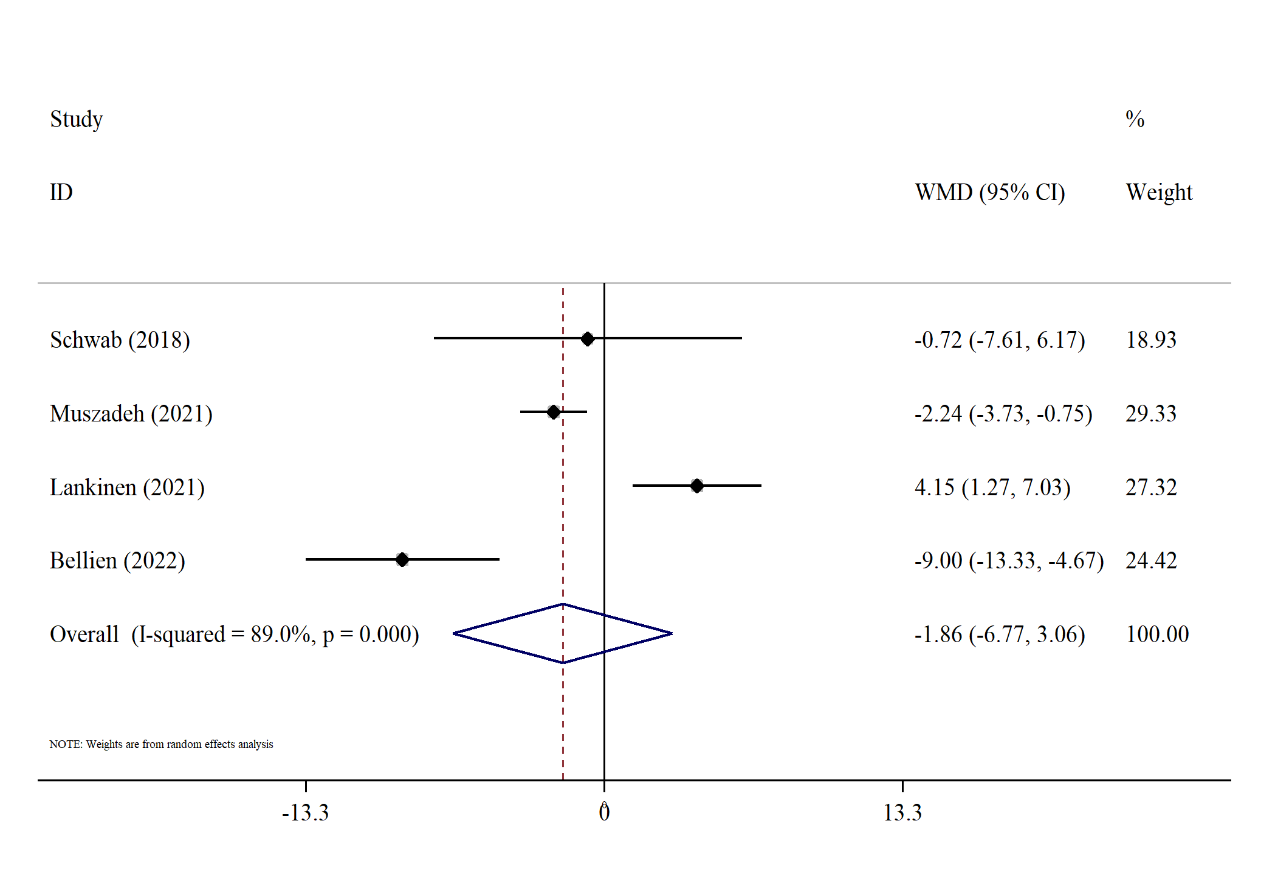 | 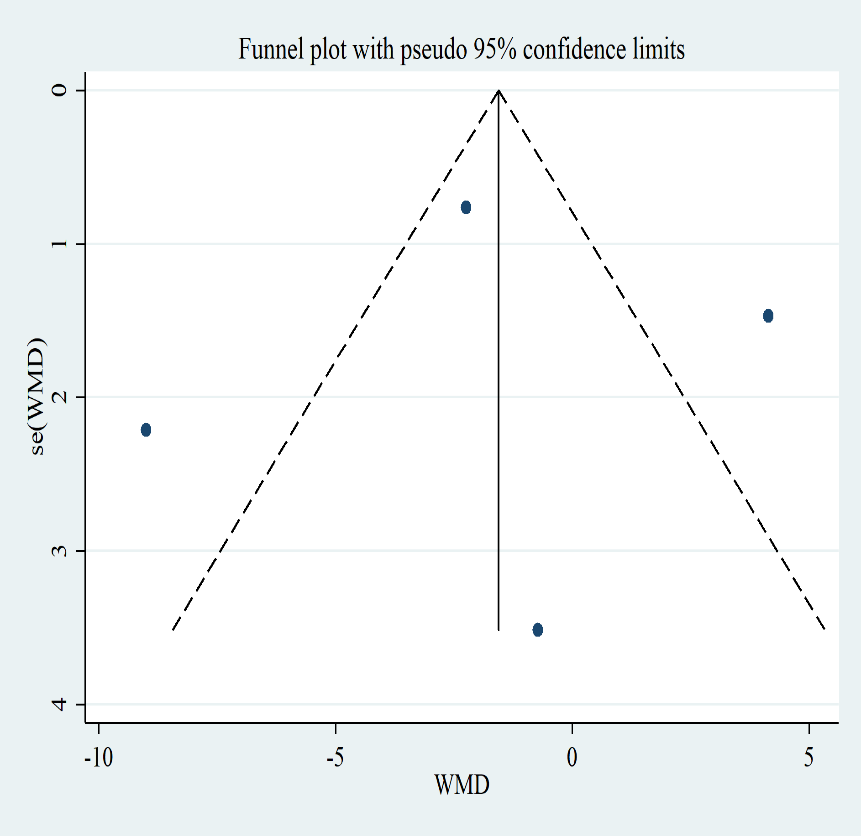 |

**Supplemental Figure 2:** Forest plot (A) and funnel plot (B) of effect of camelina oil supplementation on fasting insulin

| **Supplemental Figure 2: A** | **Supplemental Figure 2: B** |
| --- | --- |
| 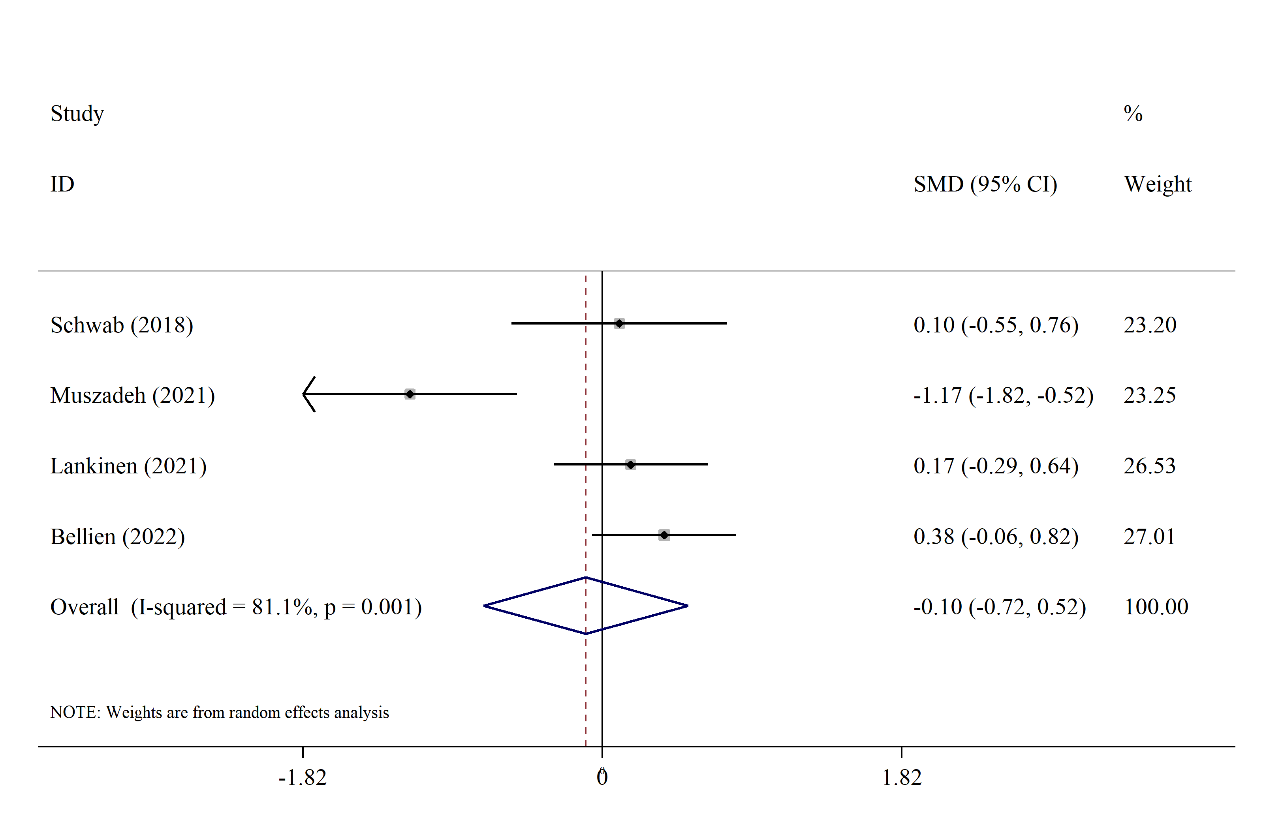 | 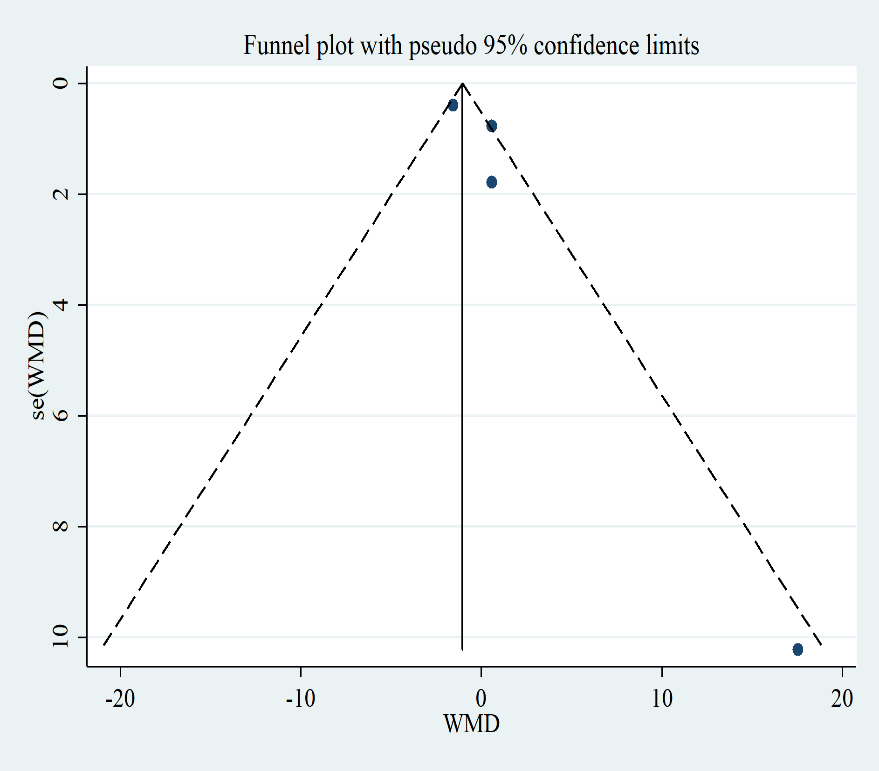 |

**Supplemental Figure 3:** Forest plot (A) and funnel plot (B) of effect of camelina oil supplementation on low-density cholesterol

| **Supplemental Figure 3: A** | **Supplemental Figure 3: B** |
| --- | --- |
| 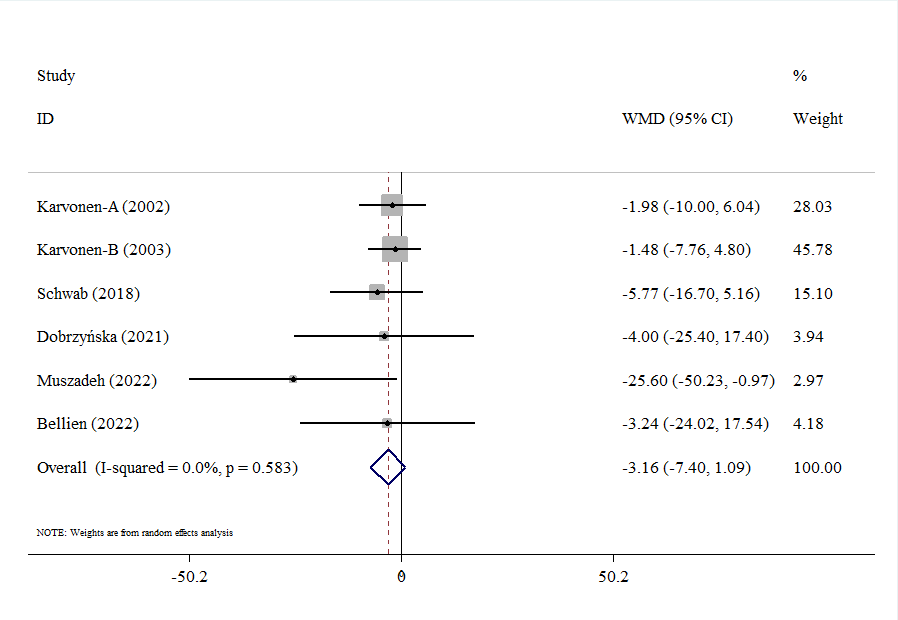 | 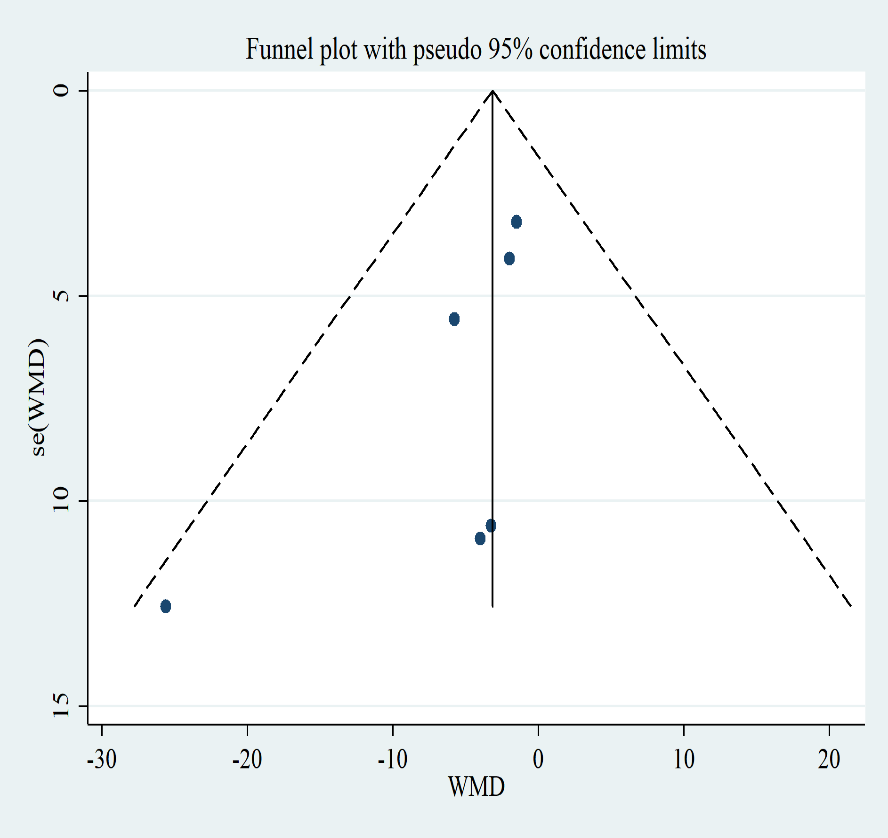 |

**Supplemental Figure 4:** Forest plot (A) and funnel plot (B) of effect of camelina oil supplementation on high-density cholesterol

| **Supplemental Figure 4: A** | **Supplemental Figure 4: B** |
| --- | --- |
| 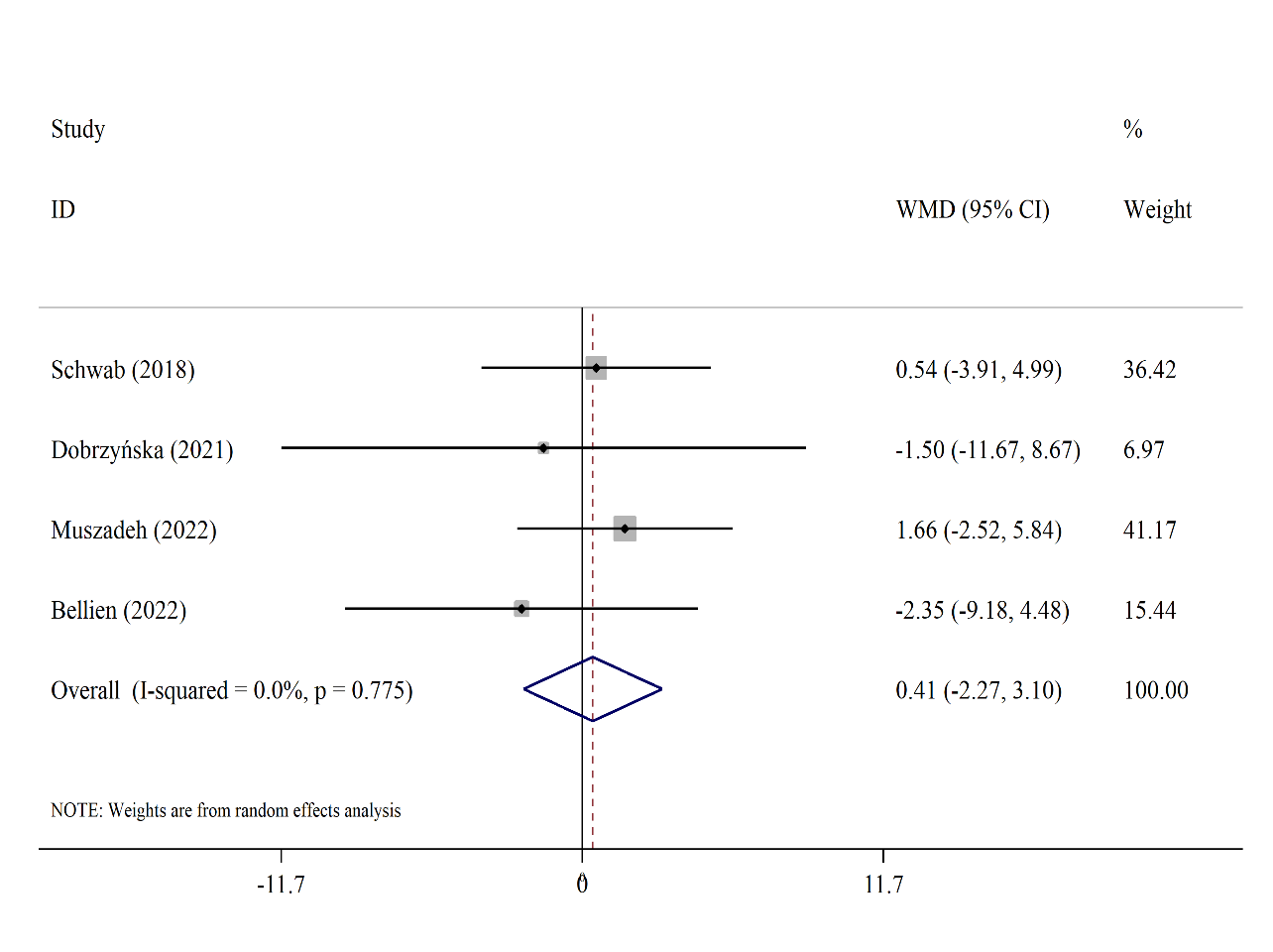 | 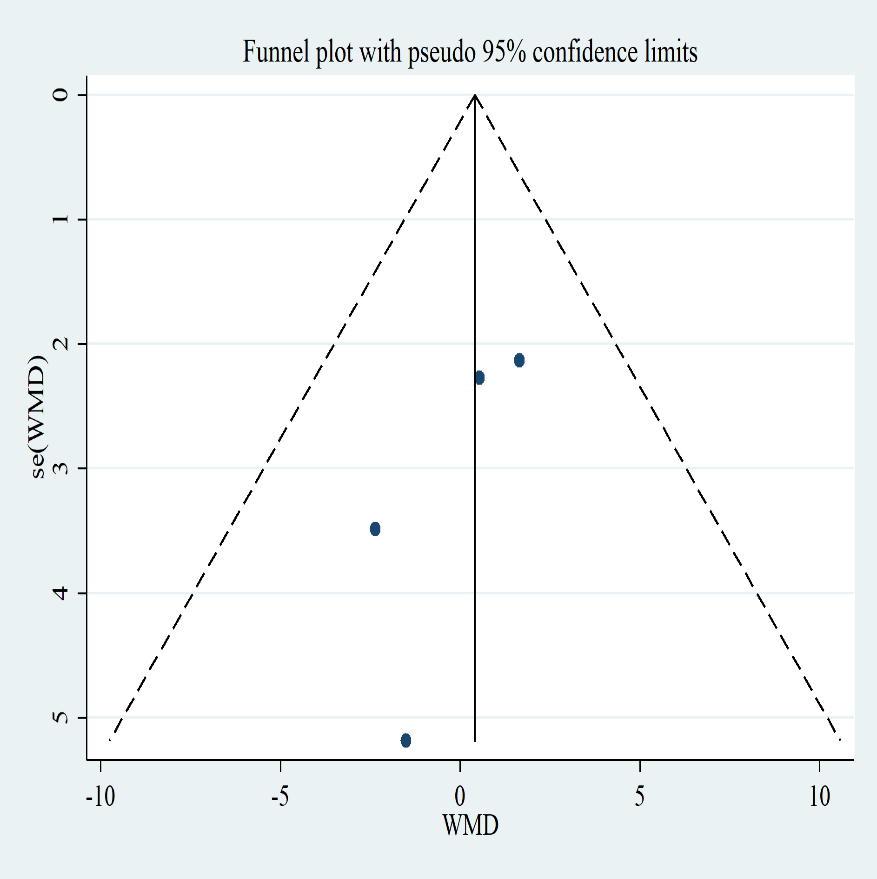 |

**Supplemental Figure 5:** Forest plot (A) and funnel plot (B) of effect of camelina oil supplementation on triglycerides

| **Supplemental Figure 5: A** | **Supplemental Figure 5: B** |
| --- | --- |
| 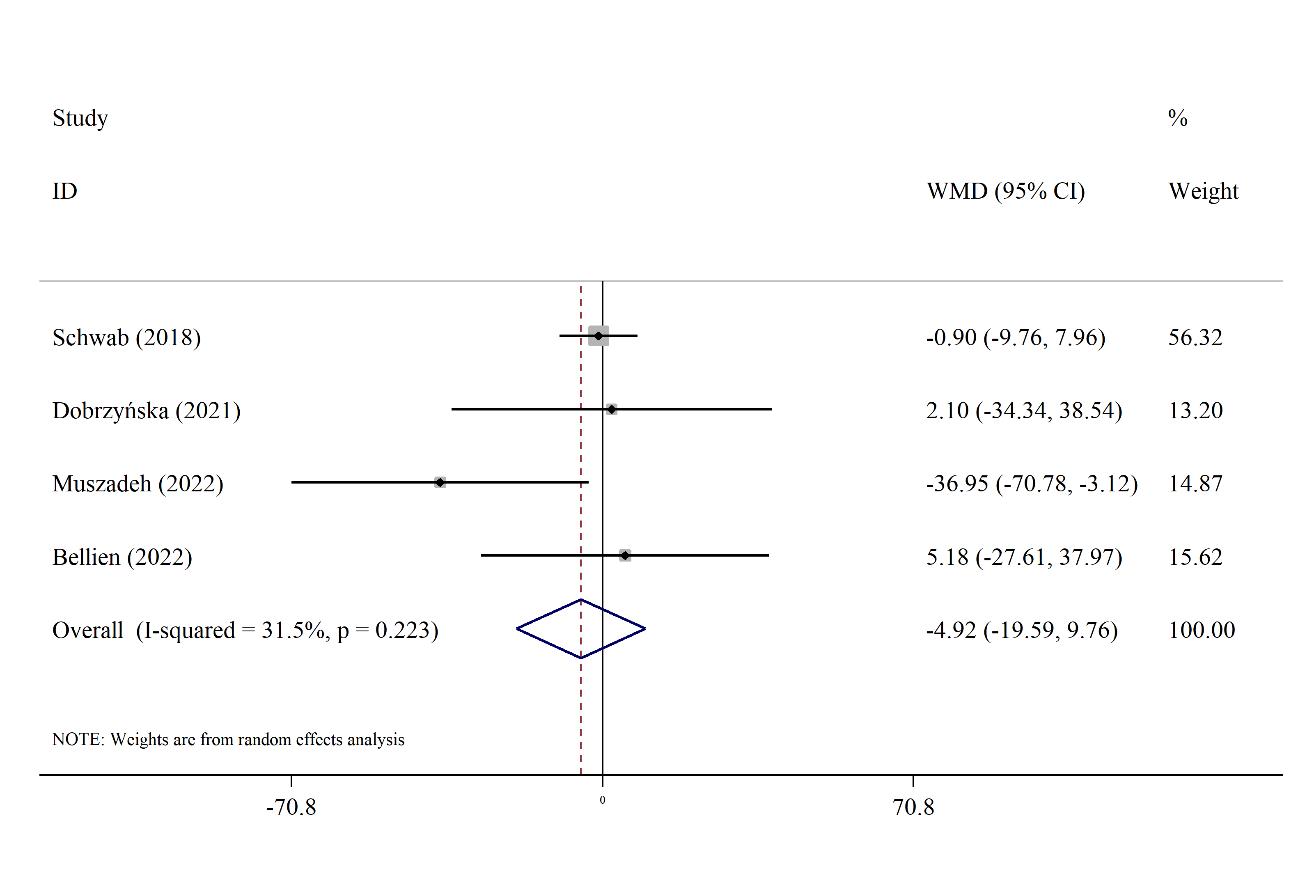 | 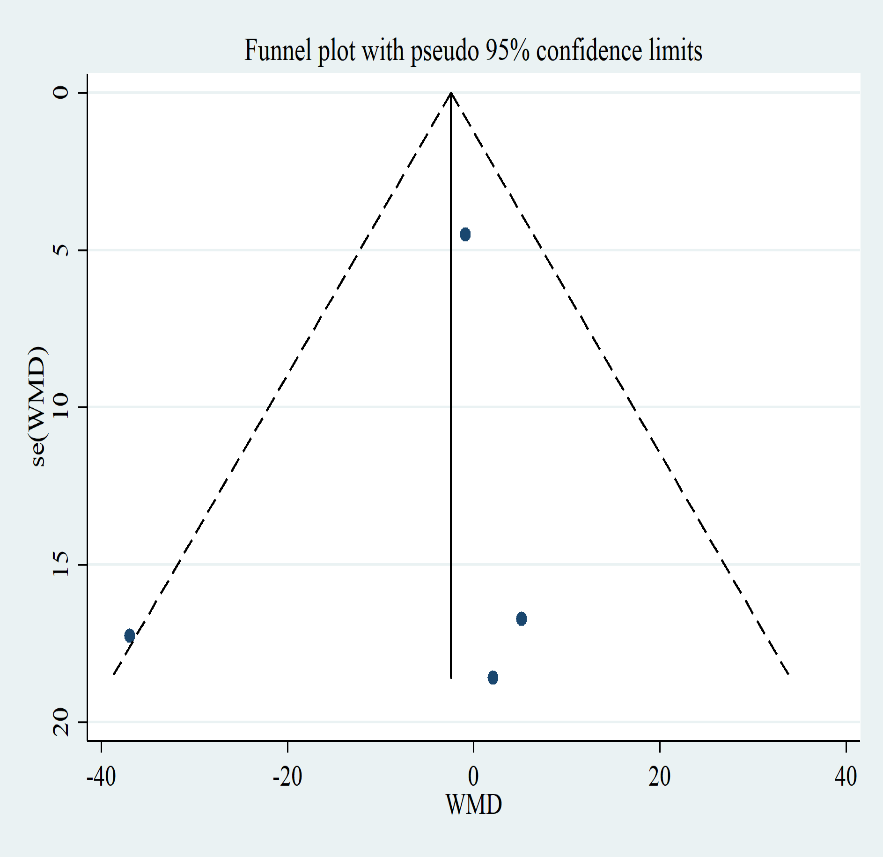 |

**Supplemental Figure 6:** Forest plot (A) and funnel plot (B) of effect of camelina oil supplementation on total cholesterol

| **Supplemental Figure 6: A** | **Supplemental Figure 6: B** |
| --- | --- |
| 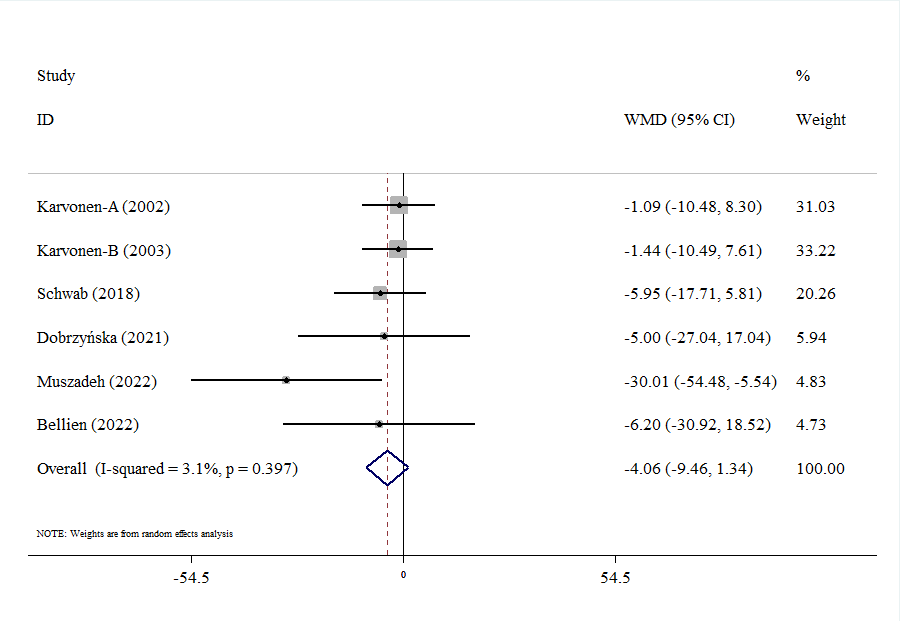 | 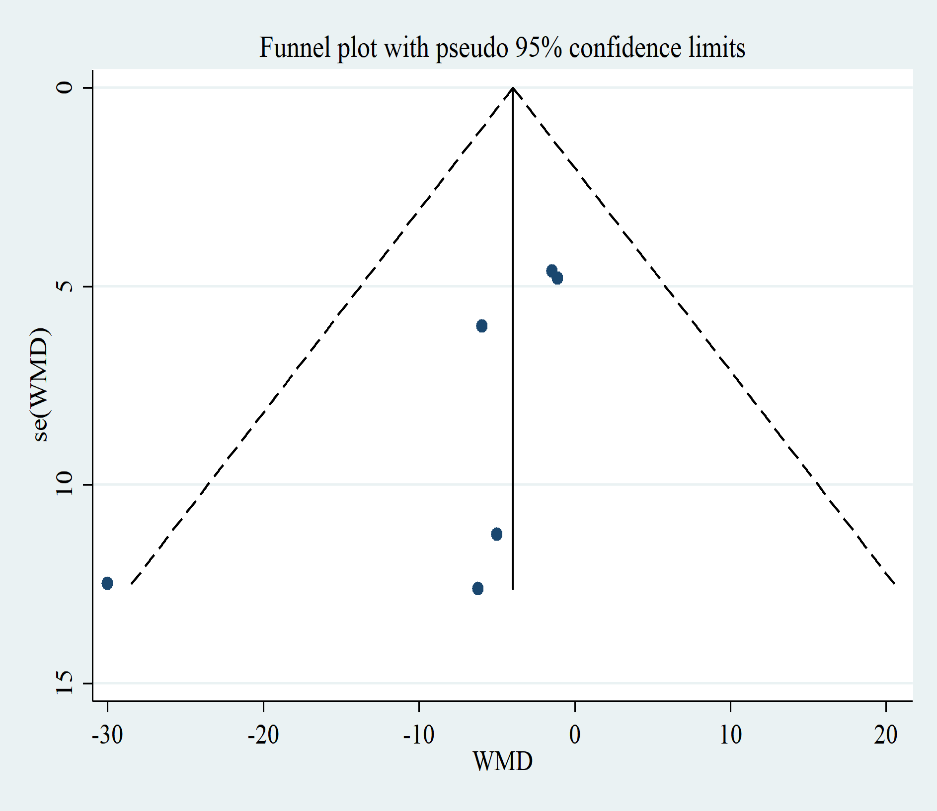 |
