# Supplementary material for: Effects of camelina oil supplementation on lipid profile and glycemic control: a systematic review and dose‒response meta-analysis of randomized clinical trials
Source: Lipids Health Dis. 2022 Dec 7;21:132. doi: 10.1186/s12944-022-01745-4 (PMC9727906; doi:10.1186/s12944-022-01745-4)
Supplement: Supplementary file 5 — Additional file 5. [file 12944_2022_1745_MOESM5_ESM.pdf]

Plagiarism Checker X Originality Report

Similarity Found: 9%

Date: Thursday, November 03, 2022
Statistics: 355 words Plagiarized / 3829 Total words
Remarks: Low Plagiarism Detected - Your Document needs Optional Improvement.

Introduction Prior research indicates that alpha-linolenic acid (18:3, n-3; ALA) can reduce the risk of cardiovascular disease (CVD) by improving blood lipids, blood pressure, and hemostatic factors, among others(1-3). According to a meta-analysis published in 2020, an increase in the intake of ALA is associated with a decrease in triglycerides (TG), total cholesterol (TC), low-density cholesterol (LDL), and very low-density lipoprotein cholesterol (VLDL) levels (1). Camelina oil (derived from Camelina sativa), a lesser-known oil, is considered a good source of ALA compared to other edible oils; 36% to 40% of its fatty acid content is ALA, an n-3 fatty acid derived from plants(4, 5).

Moreover, it is one of the richest dietary sources of omega-3 fatty acids, with a polyunsaturated fatty acids (PUFA) content of over 50%, as well as high contents of antioxidants, namely tocopherols (55.8-76.1 mg/100 g), carotenoids (103-198 mg of carotene/kg), and phytosterols (331-442 mg/100 g)(6, 7). Numerous randomized controlled trials (RCTs) were performed to assess the efficacy of Camelina oil supplementation (COS) on CVD-related markers, including lipid profile and glycemic parameters (6-12). These investigations have yielded contradictory results.

For instance, Musazadeh 2021 et al. (7) and Bellien et al. (8) revealed that COS might attenuate glycemic markers in nonalcoholic fatty liver disease (NAFLD) and hypertensive patients, respectively. However, Schwab et al. (12) showed that COS did not affect glycemic parameters among participants with impaired fasting glucose. Moreover, Musazadeh et al. (6) showed that COS improved lipid profile in NAFLD patients. Camelina oil has been suggested to modulate fatty acid synthesis and oxidation through the up-regulation of ?-oxidation gene expressions, such as peroxisome proliferator-activated receptor ? (PPAR?) and carnitine palmitoyltransferase-1 (CPT-1).

Furthermore, it has also been proposed to inhibit lipogenic gene expressions such as sterol regulatory element binding proteins (SREBPs), carbohydrate-responsive element-binding protein (ChREBP), and PPAR? (6). However, some studies did not show any significant effects of COS on lipid profile as a CVD-related marker(8, 12). Thus, research on this topic has shown mixed findings leading to a lack of consensus on the influence of COS on lipid profile and glycemic control.

There are currently no investigations to systematically assess and summarize findings on this topic, representing a knowledge gap. Therefore, a systematic review and meta-analysis of published RCTs were conducted to determine the effectiveness of COS on lipid profile and glycemic control in human studies. Experimental methods Systematic search and study selection The study's protocol was registered in the International Prospective Register of Systematic Reviews Database (CRD42021275655) and conducted according to the 2020 PRISMA guidelines (13).

An explanation of the population, intervention, comparator, and outcome (PICO) framework is reported in Supplementary Table 1. A systematic literature search was done in ISI Web of Science, Scopus, the Cochrane Library databases, and the PubMed search engine up to July 1, 2022, with no date or language limitations. The search strategy and the key terms are illustrated in Supplementary Table 2. A supplementary literature search was extended to Google Scholar with the screening of camelina oil-related terms up to October 26, 2022.

The first ten pages of all search records were scanned. Database searches were completed in conjunction with the bibliographical examination of all relevant papers. Two authors (separately) performed the systematic screening. Any disagreements were decided by a discussion with another researcher. Eligibility Criteria Two researchers screened the titles, abstracts, and full texts of relevant studies. All RCTs in humans (either parallel or cross-over designs) that evaluated the effect of COS on lipid profile (LDL, high-density lipoprotein [HDL], TG, and TC) and glycemic indices (fasting blood sugar [FBS] and fasting insulin [FI]) were selected.

The exclusion criteria included clinical trials with an intervention duration of less than two weeks, clinical trials without a placebo group and those which were not randomized, use of COS in combination with another supplement, observational or animal studies, book section, editorial, conference paper, letter, short survey, notes and those with insufficient data for the outcomes of interest. Data extraction The main features of included studies are reported in Table 1. If there was no available relevant data, corresponding authors were contacted to obtain any missing data. The data extraction procedure was conducted separately by two researchers to ensure reliability.

Any disagreements were resolved by consensus and discussion. Quality assessment of studies The Cochrane Collaboration tool (14) was applied to evaluate the quality of articles according to the following criteria: (1) random sequence generation (selection bias), (2) allocation concealment (selection bias), (3) blinding (performance bias and detection bias), (4) separated for blinding of participants and personnel, as well as blinding of outcome assessment, (5) incomplete outcome data (attrition bias), (6) selective reporting (reporting bias), and (7) other biases (any essential concerns about bias not covered in the other domains of the tool).

Each area was categorized into three levels: low risk of bias (bias, if present, is unlikely to alter the results seriously), high risk of bias (bias may alter the results seriously), and unclear risk of bias (a risk of bias that raises some doubts regarding the results). Based on these areas, the overall quality of each study was weighed as good (low risk for more than two items), fair (low risk for two items), and weak (low risk for less than two items)(15). Meta-analysis of data To assess the effect size of the lipid and glycemic markers, the mean and standard deviation (SD) changes were extracted from the COS and placebo groups.

Subgroup analyses relating to the study duration (? 8 weeks and > 8 weeks), sex (female or both), body mass (normal, overweight, or obese), dosage (< 30 g/day and ? 30 g/day), participants' baseline body mass index (? 25 and > 25) and mean age (? 55 and > 55 years) were carried out to found potential sources of heterogeneity. For the random-effects model, the DerSimonian and Laird method was applied (16). Within-group changes were calculated by subtracting the baseline mean from the final mean value in each group. The SD of the mean difference was calculated using the following formula: SD change = (17).

For trials that reported only the standard error of the mean (SEM), SD was calculated applying the following formula: SD = , where “n” represented the number of participants in each group. Heterogeneity between studies was evaluated by Cochrane's Q test (significance at $P < 0.100$) and I2 index. The potential non-linear effects of COS dose (g/d) and study duration (week) were evaluated by applying fractional polynomial modeling (18). Sensitivity analysis was done by eliminating each study one by one and recalculating the pooled assessments. Publication bias was evaluated by Egger's regression asymmetry (19).

All statistical analyses were performed utilizing STATA software, version 16 (Stata Corp LP, College Station, TX). Results were considered significant at $P < 0.05$. Certainty assessment The general certainty of evidence in randomized clinical trials was ranked

utilizing the Grading of Recommendations Assessment, Development, and Evaluation (GRADE) working group guidelines. According to the relevant evaluation criteria, the quality of evidence was ranked into four classes: high, moderate, low, and very low(20).

Results Selection and identification of studies The study's systematic literature search and study selection flow is reported in Figure 1. The systematic literature search found a total of 3782 studies, of which 2124 were evaluated (1658 articles excluded by duplication). Two thousand one hundred ten records did not meet the inclusion criteria and were excluded from qualitative and quantitative analyses.

In contrast, seven studies were excluded from the quantitative evaluation for reporting irrelevant outcomes (n=3), not presenting sufficient data (n=2), and using COS in combination with other supplementations (n=2), Supplementary Table 3. Finally, seven RCTs, collectively comprising six markers, were identified for the quantitative analysis (6-12). Characteristics of studies The seven eligible RCTs included 428 individuals (202 participants in the COS and 226 in the control group) (Table 1). The mean age of the participants ranged from 44.30 ± 4.38 to 66.6 ± 5.6 years.

Trials were conducted in Finland (10-12), Iran (6, 7), Poland (11), and France (8). Included clinical trials were conducted in healthy men (11) and hypercholesterolemic participants (10), as well as in participants with impaired fasting glucose(12), NAFLD participants (6, 7), postmenopausal women with dyslipidemia (9), and hypertensive patients with metabolic syndrome(8). All of the studies applied a parallel arm setting. These articles were published between 2002 and 2022.

The dose of COS used ranged from 50 ml/d to 30 g/d, while the length of the interventions ranged from 6 to 24 weeks. The types of interventions used for the control groups included canola oil (9, 10), sun?ower oil (6, 7, 11), a diet with limited intakes of fish and sources of ALA (12), as well as a mixture of cyclodextrin(8). Quality assessment of studies According to the outcomes of the Cochrane risk of bias tools, all clinical trials were categorized as good quality (demonstrating a low risk of bias on ? 3 domains) (6-12) (Table 2).

As illustrated in Table 2, all studies were ranked low risk for random sequence generation and allocation concealment domains(6-12). Lankinen et al.(11) was ranked at high risk for blinding participants and personnel and blinding the outcome assessors' domains. However, these bias domains were low risk for other studies (6-10, 12). The incomplete outcome data domain of bias was low risk for six studies (6-9, 11, 12), and the Karvonen et al. study (10) was ranked as having an unclear risk of bias.

Four studies were ranked low risk (6, 8, 11, 12), and three were categorized as having an unclear risk for selective reporting bias domain (7, 9, 10). All studies were ranked low risk for other bias domains (6-12). Meta-analysis of data Effects of COS on glycemic indices As shown in Table 3, pooled data from four clinical trials demonstrated that the COS did not change FBG (-1.86 mg/dl; 95% CI: -6.77, 3.06; I2 = 89.0%; P = 0.459) and FI (-0.10 pmol/L; 95% CI: -0.72, 0.52; I2 = 81.1%; P = 0.752) compared to the placebo group (Supplemental Figures 1 and 2, Part A). Effects of COS on lipid profile Data analysis from six trials that evaluated lipid profile showed that COS did not significantly change LDL (-3.16 mg/dl; 95% CI: -7.40, 1.09; I2 = 0.0%; P = 0.145; n= 6), HDL (0.41 mg/dl; 95% CI: -2.27, 3.12; I2= 0.0%; P = 0.763), TC (-4.06 mg/dl; 95% CI: -9.46, 1.34; I2= 3.1%; P = 0.141), and TG (-4.92 mg/dl; 95% CI: -19.59, 9.76; I2= 31.5%; P = 0.512) compared to the control group (Table 3 and Supplemental Figures 3 to 6, Part A). However, subgroup analysis according to duration and the dose of the intervention showed that COS significantly decreases TC (-11.64 mg/dl; 95% CI: -25.49, -2.21; I2= 35.8%; P = 0.009) (Table 4) in trials with more than eight weeks in length and dosages of less than 30 g/d.

The results of fractional polynomial modeling indicated that there were non-linear dose-response relations between the dose of COS and absolute mean differences in LDL (P = 0.024), HDL (P = 0.003), and TC (P = 0.042), but not TG (P = 0.515), (Figure 2). According to Figure 2, the greatest COS effect occurs at a dosage of 20 g/day. However, there were no relationships between the duration of COS and absolute mean differences in LDL (P = 0.250), HDL (P = 0.532), TC (P = 0.276), and TG (P = 0.515); (Figure 3). Sensitivity Analysis Sensitivity analysis was conducted by removing each of the selected trials. The outcomes revealed that the weighted mean difference (WMD) was not altered remarkably by removing each of the trials.

This showed that the meta-analysis outcomes were stable and not sensitive to any of the seven trials. Publication Bias Furthermore, no evidence of publication bias was observed for the effect of COS on FBS (P = 0.970, Egger's test), and FI (P = 0.275, Egger's test), LDL (P = 0.128, Egger's test), HDL (P = 0.128, Egger's test), TG (P = 0.200, Egger's test), TC (P = 0.117, Egger's test), (Table 1). In addition, the funnel plots were symmetrical, which showed no clear publication bias among the included studies (Supplemental Figures 1 to 6, Part B).

Quality of evidence The GRADE guidelines were utilized to evaluate the quality of evidence for the outcomes. The effects of LDL, HDL, TG, and TC were downgraded to a moderate level. Moreover, FBS and FI were categorized as very low quality (Table 5). Discussion The current meta-analytic investigation assessed the effects of COS on lipid and glycemic profiles. Pooled data analysis did not indicate any effects of COS on lipid profile and glycemic markers compared with placebo. However, subgroup analysis

showed that COS for more than eight weeks and at a dose lower than 30 g/d could decrease TC.

Furthermore, the results indicated that there were non-linear dose-response relations between the dose of COS and absolute mean differences in LDL, HDL, and TC, but not TG. The greatest COS effect occurs at a dosage of 20 g/day. Recently, nutraceutical products have gained attention for lessening CVD risk. This is important as data showed 18.6 million deaths due to CVD in 2019 alone (21). Camelina sativa L., also known as false flax, may have a beneficial effect on reducing CVD risk due to its high content of polyunsaturated fatty acids.

Because the omega-3 fatty acid is abundant in Camelina oil, and it contains 40-45 % ALA, 15% linoleic acid (LA), and a low amount of saturated fatty acids (SFA) (approximately 6%)(22), it is theorized that COS could improve CVD risk. Yet, no study has summarized previous findings on this topic. The current investigation revealed a cardioprotective impact of COS through a systematic review and meta-analysis for the first time. The analysis revealed no effect of COS on FBG and FI. In line with these results, a study performed on participants with an impaired glucose metabolism found that 12 weeks of COS did not influence glycemic markers (12).

Additionally, another study showed that an 8-week COS did not change FBS and FI compared with sunflower oil intake (11). Another study in NAFLD participants demonstrated that COS intake for 12 weeks did not alter FBG, but the intervention improved FI compared to 20 g/d sunflower oil intake (23). In contrast with the result of this study, a recent clinical trial conducted by Bellien et al.

among hypertensive patients with metabolic syndrome compared the effects of cyclodextrin-complexed camelina oil with a placebo containing cyclodextrins and wheat starch for six months and demonstrated that COS intake enhanced fasting glycemia (24). Since the study by Bellien was longer than prior investigations, it seems that long-term COS can alter glucose metabolism, and these inconsistent results in available literature may be due to the different durations of COS. Alternatively, genetic factors can affect the fatty acid composition.

For instance, delta-5-desaturase and delta-6 desaturase are limiting enzymes in the endogenous pathway of omega-3, and omega-3 biosynthesis is encoded by fatty acid desaturase- 1 and fatty acid desaturase- 2 genes. So variation and single nucleotide polymorphisms of fatty acid desaturase-1and fatty acid desaturase- 2 genes can affect the biosynthesis of PUFA(25). Moreover, a study by Lankinen et al. in participants with different FADS1 rs174550 genotypes (TT or CC) revealed that COS for eight weeks

increased FBS in men with the carrier TT genotype for FADS1 rs174550 compared to baseline values (11).

Also, a recent meta-analysis revealed that omega-3 intake increases the gluconeogenesis of glycerol (26). Consequently, chronic high-dose omega-3 treatment could negatively affect glycemic control among diabetes mellitus patients (26). Another meta-analysis of 8 clinical trials in type 2 diabetes found that ALA-enriched diets with a median of 4.4 g/d ALA did not change FBG and FI (27). There is evidence showing that the conversion rate of ALA to eicosapentaenoic acid (EPA) and docosahexaenoic acid (DHA) is low, so the beneficial effect of EPA/DHA derived from ALA intake on the glycemic profile is doubtful (28, 29).

On the other hand, in some included studies, sunflower oil was considered a placebo for COS. Sunflower oil encompasses nearly 85% unsaturated fatty acids (14-43% oleic and 44-75% linoleic acids) (30). The beneficial effect of oleic acid intake on the glycemic profile was observed previously. A prior investigation showed that oleic acid simplifies glucose uptake in adipocyte tissue by enhancing the signaling of insulin receptors (31). Furthermore, meta-analytic work by Wu et al. (32) demonstrated that a higher ratio of linoleic acid biomarkers was related to a reduced risk of type 2 diabetes, which might be related to increased insulin sensitivity(33).

Thus, it seems that if studies considered another component as a placebo, the favorable effects of COS could be better manifested. Moreover, the lack of significant changes in markers may be related to small number of studies. Hence, more RCTs are warranted to further assess glycemic indices following COS, particularly using placebo that have no favorable effect on glycemic control as well as including participants with insulin resistance. Dyslipidemia is another risk factor of CVD that plays an important role in the initiation and progression of the disease. The pooled data analysis did not demonstrate a significant change in lipid profile after COS compared to placebo. Karvonen et al.

(34) conducted a clinical trial comparing the cholesterol-lowering effects of Camelina, Rapeseed, and Olive oil (30 g/d for each) in hypercholesterolemic participants. Their findings revealed lessened cholesterol levels in all three groups compared with baseline values (34). However, they did not find a significant difference between the three groups. This study used COS at the dose of 30 g/d and for a short-term duration. It seems that a longer-time of dietary intervention may be needed to exert more noticeable cholesterol-lowering effects.

Findings of this investigation also showed that ALA (C18:3, n-3) increased significantly in the camelina oil compared to the other groups. It seems that using other oils as a

placebo could not be effective in finding between-group differences because rapeseed oil and olive oil have a cholesterol-lowering effect (35-37). Rapeseed oil contains polyphenols and high amounts of unsaturated fatty acids, mainly monounsaturated fatty acids (MUFA) that can effectively reduce cholesterol levels by enhancing the excretion of bile acid and reducing cholesterol absorption (35).

Moreover, olive oil contains approximately 55 -83% oleic acid, 4 - 20% PUFA and other components such as phenolic compounds(38). A recent meta-analysis revealed that olive oil intake decreased TG, TC, and LDL, but its effects were lower than other vegetable oils, including omega-3-rich vegetable oils. However, prior research indicates that refined olive oil could not benefit lipid profile(39). This may be due to the higher level of antioxidants and the existence of phytochemical composites in virgin olive oil compared with refined olive oil (38).

The beneficial effect of olive oil on TG levels may also be due to its high amount of MUFA. It has been shown that MUFAs reduce TG by affecting the enzymes involved in the metabolism of VLDL (40). Furthermore, a recent study conducted in postmenopausal women with dyslipidemia revealed no differences between the COS and canola oil groups in terms of lipid profile. However, TC, LDL, TG, and non-HDL decreased in both groups after the six weeks of intervention compared with its baseline value(9). This study also used canola oil for comparison, which has beneficial effects on lipid profiles.

A recent meta-analysis found that canola oil intake decreased LDL, TC, and LDL/HDL ratio compared with olive oil intake (41). Both of these oils contain high amounts of MUFA, but canola oil contains a higher amount of PUFAs, particularly ALA (42-44). The exact mechanisms for the lipid-reducing influences of canola oil have not been determined yet, but it could be owing to its fatty acid components. ALA can reduce the activity of the limiting enzyme in cholesterol synthesis, ?-Hydroxy ?-methylglutaryl-CoA (HMG-CoA) (45, 46).

Also, it can play a role in increasing the beta-oxidation of fatty acids in the mitochondria, leading to decreases in both TG synthesis and the activity of enzymes involved in fatty acid synthesis(47-49). Another recent study in hypertensive patients with metabolic syndrome did not demonstrate any beneficial effects on lipid profile after 6 months of COS (10.4 g/d) compared to a placebo intervention. However, 50% of participants in that study consumed lipid-lowering agents, which may have affected their results (24). In contrast, Scwab et al.

showed that 30 ml COS for 12 weeks improved LDL and TC compared with groups that consumed fatty fish and lean fish but not in comparison with groups that were

instructed to limit intake of fish and ALA sources (50). Also, Musazadeh et al. revealed COS decreased TC, LDL, and TG in NAFLD patients after 12 weeks compared with a placebo that contained sunflower oil, although HDL did not change (51). According to the subgroup results, TC decreased in doses of less than 30 g/d and intervention durations of more than eight weeks.

It is plausible that COS higher than 30 g/d can lead to an increase in the percentage of energy intake, as a previous study demonstrated that ALA intake higher than eight g/d increased energy intake and consequently increased the risk of metabolic syndrome(52, 53). Strengths and limitations The main strength here is that it is the first meta-analysis study that investigated the influences of COS as a nutraceutical component on indicators of CVD risk. A limitation of the present research is the small number of studies included in the analysis. This may have played a role in the lack of significant changes in some of the assessed parameters. More studies must be conducted in this field.

Additionally, studies were performed in Iran and European countries. Further studies in other areas are needed to see if outcomes apply to other ethnic cohorts. Conclusion The present meta-analysis showed that COS improved TC in studies lasting more than eight weeks and dosages lower than 30 g/d. Decreases of 39 mg/dL in TC values can diminish all-cause and coronary heart disease-related mortalities by 24% and 25%, respectively(54). Thus, the declines in TC (-11.64 mg/dl) concentrations unveiled by our analysis support the clinical significance of COS as a nonpharmacological strategy for improving this lipid marker.

In addition, the results of fractional polynomial modeling indicated that there were non-linear dose-response relations between the dose of COS and absolute mean differences in LDL, HDL, and TC but not TG. The greatest COS effect occurs at a dosage of 20 g/day. Based on the results of this study, hyper-cholestrolemic participants may benefit from long-term consumption of this oil at a dosage of less than 30 g/d, and it may be considered adjuvant therapy for them; however, more studies are needed to confirm this finding. According to the data pooled in this study, some investigations used different types of oils as a placebo. These oils can have beneficial effects on lipid profile and glycemic control, which may have influenced the results of those investigations.

Consequently, it is recommended to design further studies with a suitable placebo. Additional studies utilizing different dosages and populations are recommended to expand current findings.

INTERNET SOURCES:

<1% - https://www.careomnia.com/alpha-linolenic-acid-18-3-n-3-ala-what
<1% - https://www.ncbi.nlm.nih.gov/pmc/articles/PMC5696634/
<1% - https://www.webmd.com/diet/foods-high-in-fatty-acids
<1% - https://pubmed.ncbi.nlm.nih.gov/24567203/
<1% - https://pubmed.ncbi.nlm.nih.gov/34423525/
<1% - https://www.nature.com/articles/s41430-017-0062-1
<1% -
https://www.researchgate.net/publication/358295208_Replacement_of_fish_oil_by_camelina_and_black_soldier_fly_larvae_oils_in_diets_for_juvenile_Totoaba_macdonaldi_and_their_effect_on_growth_fatty_acid_profile_and_gene_expression_of_pancreatic_lipases
<1% - https://pubmed.ncbi.nlm.nih.gov/33096051/
<1% -
https://www.cambridge.org/core/journals/animal-health-research-reviews/article/systematic-review-and-metaanalysis-of-published-literature-on-prevalence-of-nono157-shiga-toxinproducing-escherichia-coli-serogroups-o26-o45-o103-o111-o121-and-o145-and-virulence-genes-in-feces-hides-and-carcasses-of-pre-and-periharvest-cattle-worldwide/40F1FEF53A1824A68DC76B97C7DD7A4A
<1% - https://pubmed.ncbi.nlm.nih.gov/29339215/
<1% - https://www.scalestatistics.com/pico.html
<1% -
https://www.researchgate.net/figure/Main-outlets-based-on-searches-in-ISI-Web-of-Science-Scopus-and-Google-Scholar_tbl1_343916574
<1% - https://e-cnr.org/DOIx.php?id=10.7762/cnr.2022.11.2.133
<1% -
https://www.researchgate.net/figure/List-of-papers-whose-corresponding-authors-were-contacted-We-asked-eleven-authors-to_tbl1_349897149
1% - https://ludwig.guru/s/any+disagreements+were+resolved+solved
<1% -
https://handbook-5-1.cochrane.org/chapter_8/8_11_blinding_of_participants_and_personnel.htm
<1% -
https://handbook-5-1.cochrane.org/chapter_8/table_8_5_a_the_cochrane_collaborations_tool_for_assessing.htm
<1% -
https://www.researchgate.net/figure/Quality-assessment-of-each-RCT-Three-bias-levels-high-risk-of-bias-low-risk-of-bias_fig2_345456772
<1% -
https://www.researchgate.net/figure/Depending-on-the-areas-and-the-overall-quality-of-life_tbl3_231609864

<1% -
https://www.cambridge.org/core/journals/british-journal-of-nutrition/article/effects-of-collagen-peptide-supplementation-on-cardiovascular-markers-a-systematic-review-and-metaanalysis-of-randomised-placebocontrolled-trials/D1BD51038B71D9A9BCA7880B1F6649F0
<1% -
https://www.researchgate.net/figure/Exploring-potential-sources-of-heterogeneity-for-the-INSIG2-rs7566605-association-with_fig2_38030954
<1% - https://www.coursehero.com/file/61186508/PSY-325-WEEK-4-ASSIGNMENTdocx/
<1% - https://byjus.com/maths/standard-error/
<1% - https://pubmed.ncbi.nlm.nih.gov/33417003/
<1% -
https://gonzalezgass.com/all-statistical-analyses-were-performed-using-stata-version-12-statacorp-tx/
<1% - https://www.sciencedirect.com/science/article/pii/S089543561630703X
<1% - https://www.ncbi.nlm.nih.gov/pmc/articles/PMC2335261/
<1% - https://onlinelibrary.wiley.com/doi/10.1111/jocs.16888
<1% - https://ludwig.guru/s/meet+the+inclusion+criteria
<1% -
https://www.transtutors.com/questions/the-authors-state-that-no-studies-were-excluded-from-the-review-on-the-basis-of-qual-7310569.htm
<1% - https://www.ncbi.nlm.nih.gov/pmc/articles/PMC6483123/
<1% - https://pubmed.ncbi.nlm.nih.gov/36035938/
<1% -
https://methods.cochrane.org/bias/resources/rob-2-revised-cochrane-risk-bias-tool-randomized-trials
<1% -
https://textranch.com/69388/the-results-are-illustrated-in-table/or/the-results-are-shown-in-table/
<1% - https://1library.net/article/sensitivity-analysis-results-and-analysis.zxvvn60v
<1% -
https://www.researchgate.net/figure/The-funnel-plots-were-virtually-symmetrical-suggesting-there-were-no-publication-bias-in_fig7_311337402
<1% -
https://www.researchgate.net/publication/51534195_GRADE_guidelines_5_Rating_the_quality_of_evidence_-_Publication_bias
<1% - https://pubmed.ncbi.nlm.nih.gov/34478339/
<1% -
https://www.researchgate.net/figure/Non-linear-dose-response-relations-between-duration-of-intervention-and-absolute-mean_fig4_346309609

<1% - https://thesuperhealthyfood.com/what-vegetables-have-omega-3-fatty-acids/
<1% - https://www.ncbi.nlm.nih.gov/pmc/articles/PMC8713091/
<1% - https://academic.oup.com/ajcn/article-abstract/115/3/694/6428398
<1% - https://www.sciencedirect.com/topics/engineering/fatty-acid-composition
<1% - https://www.ncbi.nlm.nih.gov/pmc/articles/PMC4728637/
<1% - https://www.researchgate.net/publication/340614232_Polymorphisms_in_Fatty_Acid_Desaturase_2_Gene_Are_Associated_with_Milk_Production_Traits_in_Chinese_Holstein_Cows
<1% - https://www.frontiersin.org/articles/10.3389/fnut.2022.809311/full
<1% - https://www.ncbi.nlm.nih.gov/books/NBK209320/
<1% - https://www.centrafoods.com/blog/sunflower-oil-good-or-bad
<1% - https://www.sciencedirect.com/topics/pharmacology-toxicology-and-pharmaceutical-science/oleic-acid
<1% - https://academic.oup.com/ejcts/advance-article/doi/10.1093/ejcts/ezac107/6534099
<1% - https://pubmed.ncbi.nlm.nih.gov/34900035/
<1% - https://www.express.co.uk/life-style/health/1677467/high-cholesterol-rapeseed-oil-reduces-cholesterol
<1% - https://www.researchgate.net/publication/323057040_Comparison_of_blood_lipid-lowering_effects_of_olive_oil_and_other_plant_oils_A_systematic_review_and_meta-analysis_of_27_randomized_placebo-controlled_clinical_trials
<1% - https://www.researchgate.net/publication/266796336_Comparison_of_the_quality_of_two_classes_of_olive_oil_Extra_virgin_and_refined_oil
<1% - https://pubmed.ncbi.nlm.nih.gov/22863639/
<1% - https://www.frontiersin.org/articles/10.3389/fendo.2022.876775/full
<1% - https://www.ncbi.nlm.nih.gov/pmc/articles/PMC3746113/
<1% - https://pubmed.ncbi.nlm.nih.gov/15821374/
<1% - https://study.com/learn/lesson/fatty-acid-oxidation-pathway-mechanism-what-is-fatty-acid-oxidation.html
<1% - https://www.coursehero.com/file/p129o4np/Thus-it-is-plausible-that-some-people-can-sustain-higher-self-esteem-than-others/
<1% - https://www.coursehero.com/file/p2t30ko/Strengths-and-limitations-of-the-current-study-Our-study-has-two-key-strengths/

<1% - https://www.sciencedirect.com/science/article/pii/S0009926022002227
